# Archaerhodopsin 3 is an ideal template for the engineering of highly fluorescent optogenetic reporters†

**DOI:** 10.1039/d4sc05120c

**Published:** 2024-11-18

**Authors:** Krystyna Herasymenko, Danushka Walisinghe, Masae Konno, Leonardo Barneschi, Isabelle de Waele, Michel Sliwa, Keiichi Inoue, Massimo Olivucci, Stefan Haacke

**Affiliations:** a University of Strasbourg, CNRS, IPCMS 23 Rue du Loess 67034 Strasbourg France stefan.haacke@ipcms.unistra.fr; b Department of Chemistry, Bowling Green State University Bowling Green OH 43403 USA molivuc@bgsu.edu; c The Institute for Solid State Physics, University of Tokyo 5-1-5 Kashiwano-ha Kashiwa Chiba 277-8581 Japan inoue@issp.u-tokyo.ac.jp; d Dipartimento di Biotecnologie, Chimica e Farmacia, Università di Siena I-53100 Siena Italy; e LASIRE, Université de Lille, CNRS 59000 Lille France; f LOB, CNRS, INSERM, École Polytechnique, Inst. Polytechnique de Paris 91120 Palaiseau France

## Abstract

Archaerhodopsin-3 (AR-3) variants stand out among other rhodopsins in that they display a weak, but voltage-sensitive, near-infrared fluorescence emission. This has led to their application in optogenetics both in cell cultures and small animals. However, in the context of improving the fluorescence characteristics of the next generation of AR-3 reporters, an understanding of their ultrafast light-response in light-adapted conditions, is mandatory. To this end, we present a combined experimental and computational investigation of the excited state dynamics and quantum yields of AR-3 and its DETC and Arch-5 variants. The latter always display a mixture of all-*trans*/15-*anti* and 13-*cis*/15-*syn* isomers, which leads to a bi-exponential excited state decay. The isomerisation quantum yield is reduced more than 20 times as compared to WT AR-3 and proves that the steady-state fluorescence is induced by a single absorption photon event. In wild-type AR-3, we show that a 300 fs, barrier-less and vibrationally coherent isomerization is driven by an unusual covalent electronic character of its all-*trans* retinal chromophore leading to a metastable twisted diradical (TIDIR), in clear contrast to the standard charge-transfer scenario established for other microbial rhodopsins. We discuss how the presence of TIDIR makes AR-3 an ideal candidate for the design of variants with a one-photon induced fluorescence possibly reaching the emission quantum yield of the top natural emitter neorhodopsin (NeoR).

## Abstract 2

The mixed covalent/charge transfer character of the excited state of AR-3 allows to introduce an isomerization-blocking excited state barrier, ideal for engineering fluorescent mutants.

## Graphical abstract

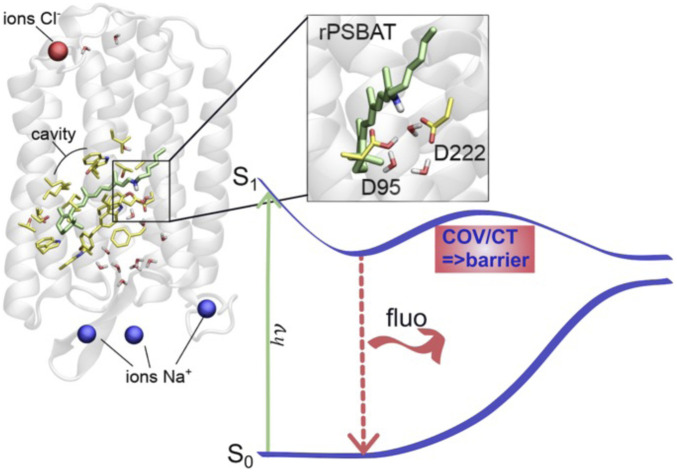

## 1 Introduction

In spite of its tiny fluorescence quantum yield (*Φ*_f_), archaerhodopsin-3 (AR-3) from the halobacterium *Halorubrum sodomense*, has been successfully used as fluorescent genetically encoded voltage indicator (GEVIs), thus enabling the visualization of neuronal activity with spatiotemporal precision.^1–4^ To enhance the relatively low *Φ*_f_ of AR-3 and expand its applicability as GEVI, several AR-3 variants were designed over the past years by screening libraries with randomly mutated AR-3.^5–8^ However, the highest *Φ*_f_ reported for the seven-fold mutated ARCH-7 is only 1.2%,^5^ still much lower than the *ca.* 20% value recently found for the exceptional naturally fluorescent neorhodopsin (NeoR).^9,10^ There is therefore a need to understand if AR-3 can be engineered to generate variants approaching or overcoming, the NeoR limit or if, alternatively, there are fundamental reasons why this is not possible.

**Fig. 1:**
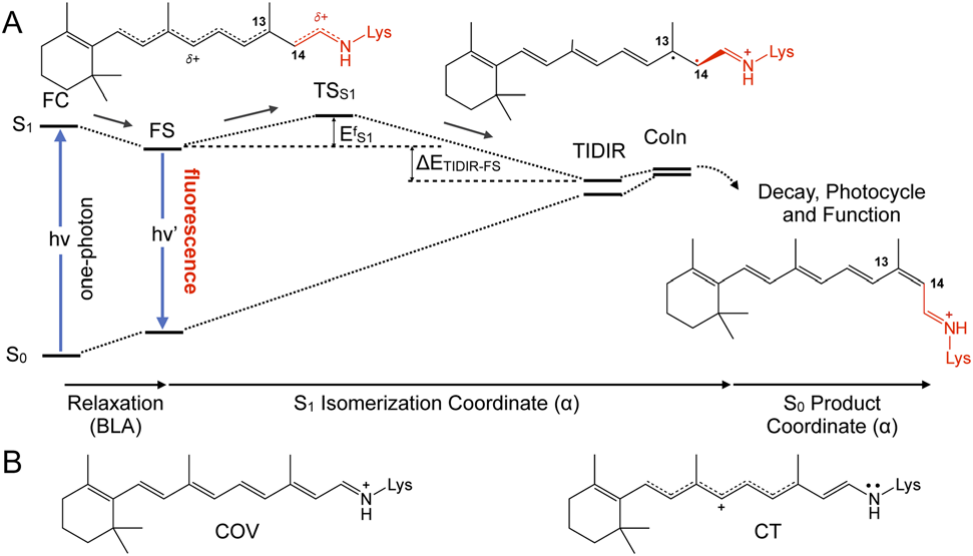
(A) Representation of the chromophore isomerization path. FS corresponds to the fluorescent state. TIDIR represents the photoisomerization channel located near CoIn. Lewis formulas representing product, FS and TIDIR display distinct degrees of double bond twisting and charge transfer. (B) Representation of electronic characters of the chromophore COV *vs.* CT. Note the different bond length alternation (BLA).

A first step towards an understanding of AR-3 was achieved by some of us *via* a theoretical/computational study of the electronic structure of the first excited state (S_1_) of its retinal protonated Schiff base (RPSB) chromophore (and fluorophore).^11^ A schematic representation of the established fluorescence mechanism in AR-3 mutants is given in Fig. 1. A potential energy surface (PES) barrier along the S_1_ isomerization path of RPSB was found to control the lifetime of the fluorescent state (FS) and prevent its efficient decay into the region of an S_1_/S_0_ conical intersection (CoIn). By computing the height of the barrier 
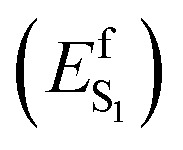
 for different AR-3 mutants, a correlation was established between barrier height and mutation-dependent *Φ*_f_.^11^ This result indicated that: (i) the higher fluorescence of randomly engineered variants is due to a 1-photon excitation process that is ineffective in WT AR-3 (ref. 12 and 13) and (ii) the increase in 1-photon *Φ*_f_ correlates with the stability (*E*_TIDIR-FS_) of an unusual S_1_ twisted bi-radical (TIDIR) of RPSB displaying a covalent (COV) electronic character, in stark contrast to the dominant charge transfer (CT) character of the S_1_ state in other microbial rhodopsins.^14–19^ Remarkably, in a more recent computational study the same mechanism was shown to be responsible for the record *Φ*_f_ of NeoR.^10,20^

Two central questions remain to be answered to establish WT AR-3 as a suitable starting point for fluorescent GEVI engineering. The first is whether the transient TIDIR intermediate is also a characteristic of WT AR-3 in spite of a substantial absence of 1-photon fluorescence in this species (WT AR-3 fluorescence originates from a complex 3-photon process).^12,13^ The second is whether it is possible to provide direct or indirect experimental evidence for the existence of TIDIR. Experimental studies on the fluorescence lifetimes of QuasAr1, QuasAr2 and Archon1 and its mutants were recently reported,^21–23^ confirming that the fluorescence is induced by a 1-photon process. Importantly, in contrast with the fact that light-adapted (LA) WT AR-3 contains a single all-*trans* chromophore isomer, the chromophore of the variants mentioned above was found to correspond to a mixture of all-*trans* and 13-*cis* RPSBs.^22,23^ For Archon1, it was also argued that the trans-membrane voltage acts on the ground isomer composition, and hence on the *Φ*_f_.^22^

In the present paper, we address the two questions above *via* a combination of spectroscopic and non-adiabatic molecular dynamics studies. Experimentally, we study the transient spectroscopy and photoisomerization efficiency of WT AR-3 and, additionally, of two specific AR-3 mutants: the double mutant DETC (D95E, T99C) and the five-fold mutant ARCH-5 (V59A, P60L, D95E, T99C, P196S) initially reported by McIsaac *et al.*^5^ For WT AR-3 at pH6, the excited state lifetimes (*τ*_S_1__)and isomerization quantum yield (*Φ*_iso_) were measured for the first time, providing indirect evidence for an excited state barrier 
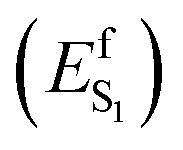
 induced by the TIDIR. Computationally, we focus on the simulation of the photoinduced dynamics of the WT AR-3 using a set of 200 quantum-classical trajectories. The results consistently support the existence of a TIDIR intermediate indicating that the mechanism illustrated in Fig. 1 is general and valid for WT AR-3, its variants as well as for NeoR. Accordingly, one concludes that the problem of finding highly fluorescent AR-3 variants, namely by increasing the average excited state lifetime 〈*τ*〉 of the system, can be solved by finding mutations that sufficiently destabilize TIDIR with respect to FS. This conclusion is indirectly supported by the spectroscopic data of the two AR-3 mutants, as we report that the 〈*τ*〉 dramatically increases to 29 and 66 ps for DETC and ARCH-5, respectively, and that the *Φ*_iso_ is reduced by more than 20 times.

## 2 Experimental results and discussion

### 2.1 Retinal configuration analysis in dark- and light-adapted proteins determined by HPLC

**Fig. 2:**
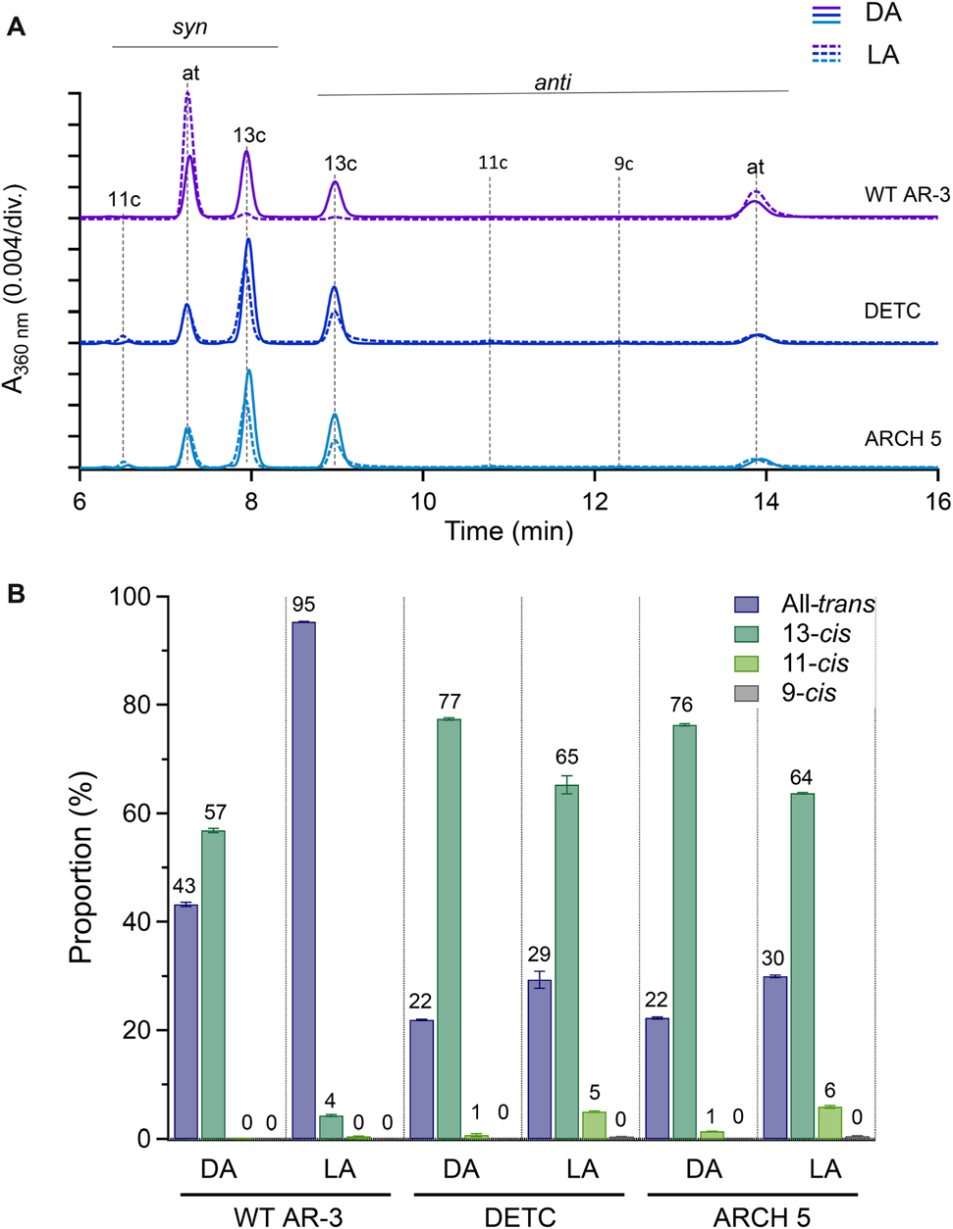
HPLC analysis of the retinal chromophore of AR-3 WT, DETC and ARCH-5 in the dark-adapted (DA) and the light-adapted (LA) states. (A) Representative HPLC chromatogram of the retinal oxime in dark-adapted (DA, solid line) and light-adapted (LA, dotted line) states. The retinal oxime was produced by the hydrolysis reaction between the retinal chromophore and hydroxylamine. *Syn* and *anti* refer to the configuration of the C=N bond of the product retinal oxime, which does not represent the configuration of the retinal Schiff base in the original proteins. The retention time of each isomer was normalized based on that of each isomer in DA. (B) The ratio of each isomer (mean ± standard deviation, *n* = 3 independent experiments). Sample illumination and HPLC analysis were performed three times independently. The numbers at the top of each bar indicate the composition of each isomer.

High-performance liquid chromatography (HPLC) analysis was used to investigate the isomeric configurations of the RPSB in light- and dark-adapted forms of WT AR-3 and the two mutants (*vide supra*). The results are given in Fig. 2. For the light-adaptation procedure, details are given in Fig. S8† together with the common associated red-shifts of absorption spectra, indicating accumulation of all-*trans* RPSB. In all proteins, the retinal primarily existed in all-*trans*/15-*syn* (AT) and 13-*cis*/15-*anti* (13-C) forms. In the dark-adapted state, 43 and 57% of WT AR-3 had AT and 13-C retinal, respectively, whereas 95% of the chromophore was in the AT form in the light-adapted state like in bR.^24^ For DETC and ARCH-5, the dark-adapted state contained ∼80% of the retinal in the 13-C form. Upon light adaptation, although a slight increase in the AT form was observed, but the 13-C remains in majority, accounting for ∼63% and ∼65% of the retinal in DETC and ARCH-5, respectively.

### 2.2 Raman spectroscopy of light-adapted proteins

**Fig. 3:**
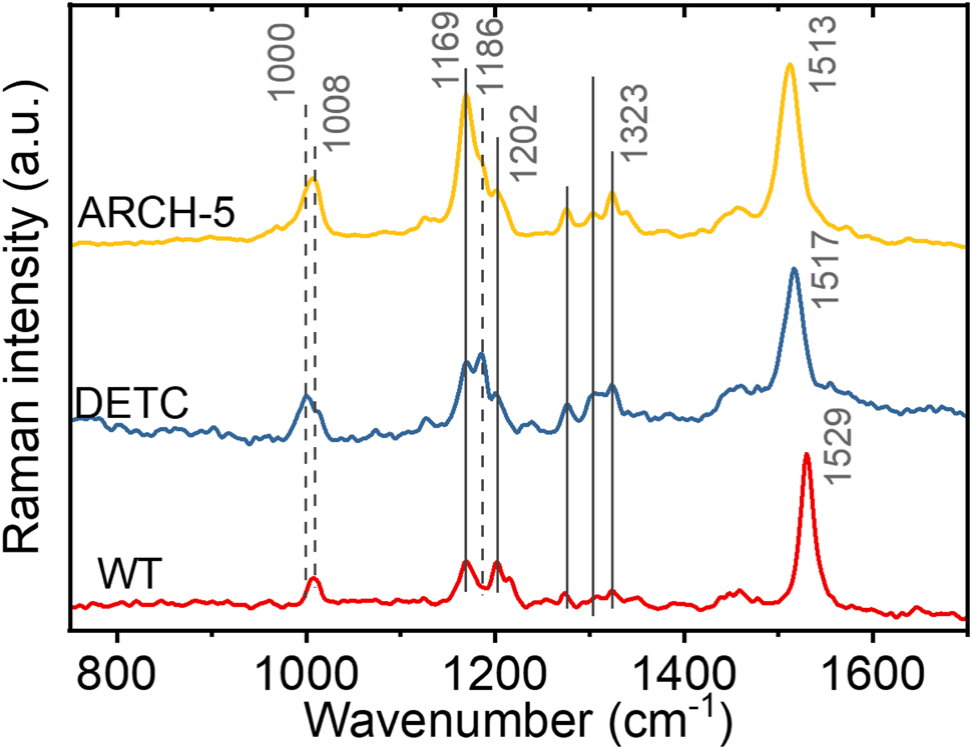
FT-Raman spectroscopy. Comparison of the off-resonance FT-Raman spectra of light-adapted WT AR-3, DETC and ARCH-5 proteins at pH 6. The positions of the common bands are highlighted with solid vertical lines. The positions of the peaks that shifted or were not present in one of the proteins are indicated by the dashed lines.

To investigate the retinal isomer composition in the LA WT AR-3, DETC and ARCH-5 Fourier transform Raman spectroscopy was used. Light adaptation was performed with the same protocol as for the HPLC experiments above (Fig. S8†). Fig. 3 presents the obtained Raman spectra for each of the proteins. For the fingerprint region (C–C single bond stretching) we can see that the WT has predominantly all-*trans* configuration of the retinal chromophore (1169 cm^−1^ and 1202 cm^−1^ bands).^25^ On the other hand, in DETC, a peak at 1186 cm^−1^, previously assigned to the 13-*cis*, 15-*syn* configuration,^25,26^ is observed. In ARCH-5, both peaks are unresolved, which could indicate a slight red-shift of the 13-*cis*-related vibration in this mutant. Moreover, in the methyl group rocking region around 1000 cm^−1^, WT and ARCH-5 display a single peak at 1008 cm^−1^, while DETC shows an additional peak at 1000 cm^−1^, which could also be a sign of the existence of two isomers. However, in the hydrogen-out-of-plane (HOOP) region around 800 cm^−1^ that was previously reported to be an indicator of a distorted 13-*cis* isomer^26^ no peak is observed for any of the three proteins. In addition, the red-shift of the C=C double frequency (from 1529 cm^−1^ in WT to 1513 cm^−1^ in ARCH-5) is observed for mutants, in line with the red shift of the mutants' absorption spectra with respect to the WT.

In conclusion, the isomer composition of the light-adapted proteins determined by Fourier transform Raman spectroscopy is qualitatively in agreement with the more precise and quantitative HPLC method (*vide supra*), with the additional information that the 13-*cis*, 15-*syn* isomer appears to be planar since no HOOP activity is observed.

### 2.3 Excited state lifetimes

**Fig. 4:**
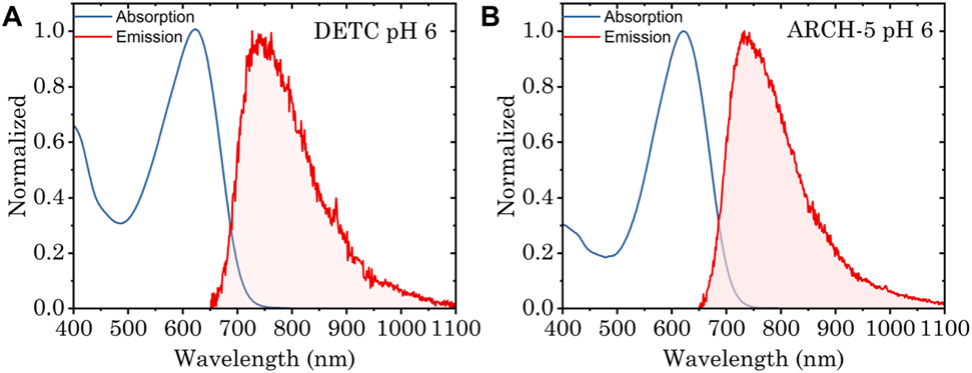
(A) Normalized absorption (blue) and emission (red) spectra of DETC at pH 6, excitation wavelength 615 nm. (B) Normalized absorption (blue) and emission (red) spectra of ARCH-5 at pH 6, excitation wavelength 600 nm.

To study the impact of point mutations on the *in vitro* fluorescence properties, the *Φ*_f_ and fluorescence lifetimes were studied for DETC and ARCH-5 in their LA forms. The absorption and emission spectra of these mutants were measured (Fig. 4). These are known to be red-shifted with respect to the WT.^5^ Moreover, *Φ*_f_ for DETC and ARCH-5 at pH 6 were calculated as described in Methods.† The obtained values of 3.6 ± 0.6 × 10^−3^ and 9 ± 1 × 10^−3^ for DETC and ARCH-5 respectively are in agreement with the ones previously obtained for slightly lower pH values.^5^

**Fig. 5:**
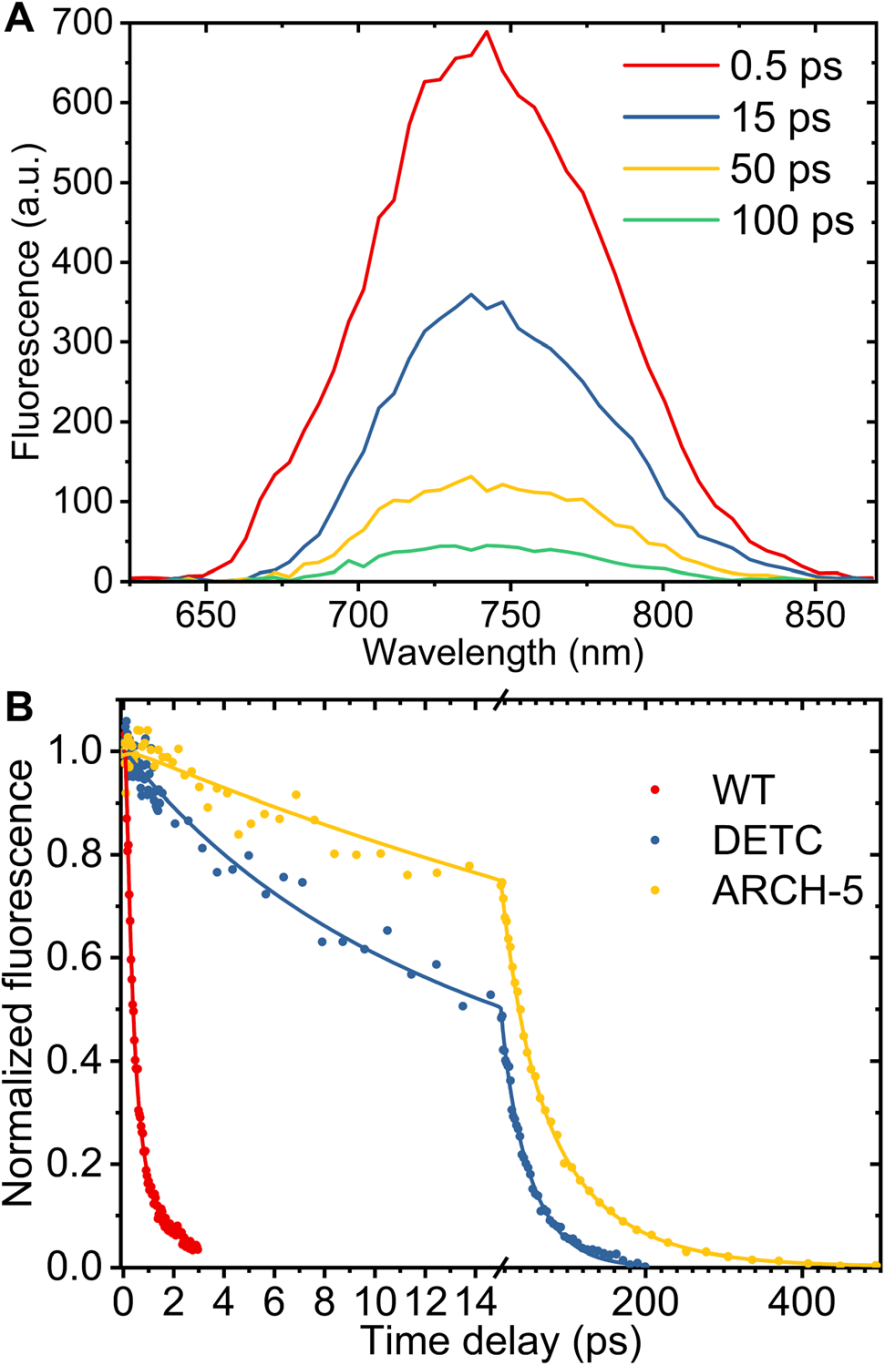
Results of FLUPS experiments. (A) Fluorescence spectra of LA AR-3 mutant DETC at pH 6 at time delays listed in the legend and for excitation at 570 nm. The difference in the fluorescence profile between steady-state emission and FLUPS measurements is caused by the phase matching conditions adjusted for up-conversion of the blue part of the emission spectrum. (B) Comparison of the normalized fluorescence decays between WT, DETC and ARCH-5 (dots). Kinetics were averaged over 15 nm around the fluorescence maximum. Solid lines present the best result obtained for fit with a bi-exponential decay function.

Broadband fluorescence up-conversion spectroscopy (FLUPS)^27,28^ with ∼150 fs time resolution (see Methods†) was used to investigate the fluorescence lifetimes of LA WT AR-3 and the two mutants at pH 6. Fig. 5A presents an example of the time evolution of DETC fluorescence spectra. According to this figure, the spectral shape does not change with time, only the fluorescent intensity decays. This indicates that the excited state dynamics do not depend on the detection wavelength and that both single and integrated kinetic analyses are relevant.

For an analysis of the kinetics, a spectral range of 15 nm around the fluorescence maximum (WT 722 nm, DETC and ARCH-5 735 nm) was averaged. The panel B of Fig. 5 presents experimental data (dots) for normalized kinetic traces for WT AR-3 (blue) and its mutants DETC (red) and ARCH-5 (yellow). It is clearly seen, that the WT fluorescence decays in a few ps while the mutants' fluorescence lives significantly longer. The kinetics have bi-exponential decay character for all the samples (Fig. S2†). Solid lines display the results of the multi-exponential fit. A Gauss function (representing the instrument response function) convoluted with two decaying exponents was used as a fitting function. A three-exponential fit was used for ARCH-5 kinetics to take into account the 0.4 ps rise of the fluorescence signal. Extracted decay time constants and their relative amplitudes are condensed in Table 1 along with calculated average fluorescence lifetimes for each of the proteins. The amplitude-weighted average fluorescence lifetimes for DETC and ARCH-5 are 29 ps and 66 ps, respectively. This approach is explained and justified in the ESI (Section 2.2†). Note that they are proportional to the above-determined *Φ*_f_ of these proteins.

**Table 1:** Fluorescence lifetimes, its relative amplitudes, amplitude-weighted average fluorescence lifetimes, measured fluorescence quantum yields and calculated non-radiative (*k*_nr_) and radiative (*k*_r_) rates of WT AR-3 and mutants at pH 6

Sample	*τ*_1_, ps	*A*_1_, %	*τ*_2_, ps	*A*_2_, %	〈*τ*_fluo_〉, ps	*Φ*_f_	*k*_nr_, ns^−1^	*k*_r_, ns^−1^
AR 3 pH 6	0.34 ± 0.02	85 ± 2	1.4 ± 0.1	15 ± 2	0.5		2000	
DETC pH 6	7.7 ± 1.1	37 ± 6	42 ± 3	63 ± 6	29	3.6 ± 0.6 × 10^−3^	34	0.12
ARCH-5 pH 6	31 ± 8	43 ± 9	91 ± 15	56 ± 3	66	9 ± 1 × 10^−3^	15	0.14

**Fig. 6:**
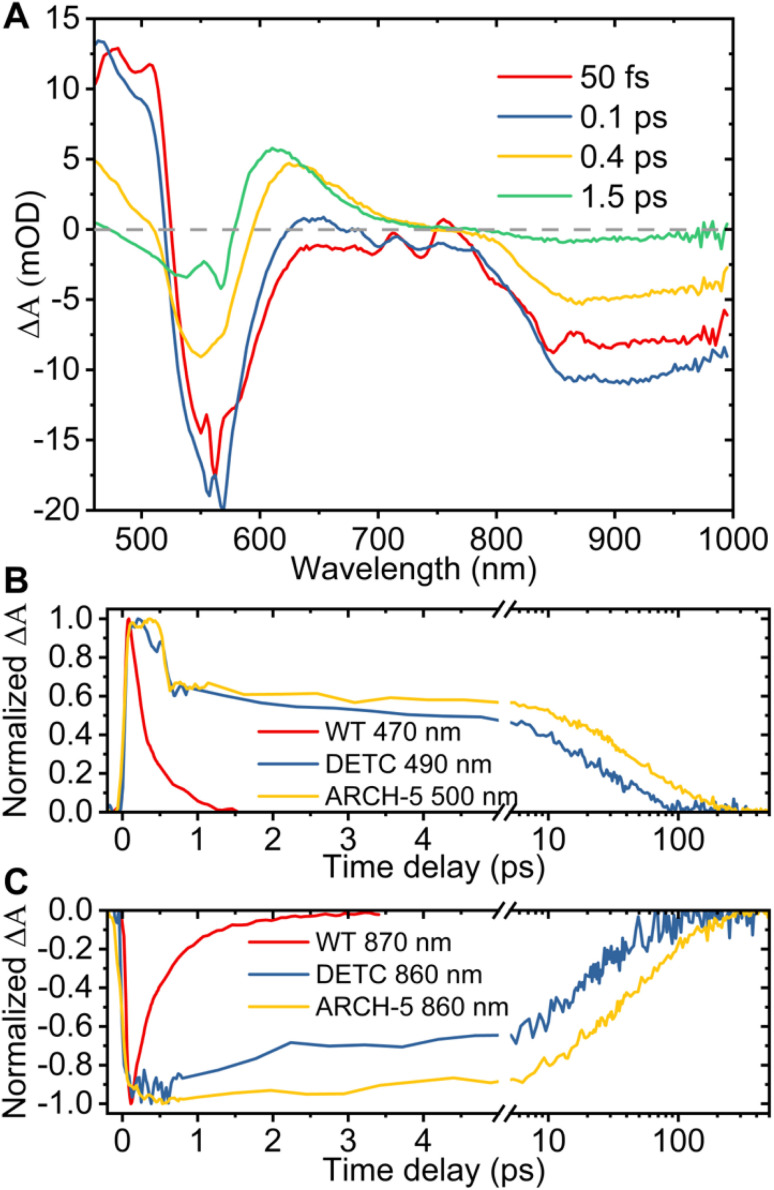
Results of TAS experiments. (A) Difference absorption spectra of WT AR-3 at the time delays indicated in the legend after the 570 nm photoexcitation. Comparison of the normalized excited state absorption (B) and stimulated emission (C) kinetics at the indicated wavelengths for WT, DETC and ARCH-5 at pH 6.

Additionally, to investigate the photoisomerization reaction and dynamics in the excited state of the proteins, ultrafast transient absorption spectroscopy (TAS) experiments were performed for the three proteins in their LA forms. In this method, the absorption changes in the 470–1000 nm range were monitored with 60 fs resolution after photoexcitation. The results of the TAS experiments are shown in Fig. 6. Panel A presents the time evolution of the difference absorption spectra of WT AR-3 (results for DETC and ARCH-5 are presented in the ESI†). We observe difference spectra typical for microbial retinal proteins, such as bR,^29–32^ highlighting excited state decay and isomerization on a sub-picosecond time scale. Indeed, at 50 fs the positive peak around 470–530 nm is due to excited state absorption (ESA) and the negative bands around 550 nm and 800–1000 nm range are assigned to the ground state bleaching (GSB) and stimulated emission (SE), respectively. Noise in the GSB region at 570 nm is caused by residual pump laser scattering. In the first 100 fs range ultrafast relaxation out of the Frank–Condon region takes place which explains the blue shift of the ESA and rise of SE in the near-IR. Later, along with the decay of the excited state (decay of ESA and SE) a new positive band appears around 630 nm. It reflects the all-*trans* to 13-*cis* isomerization of retinal, forming the vibrationally hot J intermediate, which in the next ps cools down (blue shift of its absorption to 610 nm) leading to the first ground state photoproduct of AR-3 (K intermediate).

The bottom part of Fig. 6 shows the comparison of the normalized ESA (panel B) and SE (panel C) dynamics of WT (red), DETC (blue) and ARCH-5 (yellow). Similar to the FLUPS results, DETC and ARCH-5 have significantly longer-lived excited states. At the short times (before 0.5 ps) the excited state kinetic traces of the mutants show an unusual sudden drop at 0.6 ps, which is due to excited state relaxation from the Franck–Condon region, but without the loss of its excited state population. Indeed, the stimulated emission remains constant during this time interval (see the complete time-dependent spectra for DETC and ARCH-5 in ESI,† panels A of Fig. S3 and S5†). In contrast with the WT, only a small absorption trace of photoproduct formation is detected for DETC and even weaker so for ARCH-5 for delays of hundreds of picoseconds, *i.e.* after excited state decay (Fig. S6†). The TAS results were globally fitted with three time constants. Results of the fit are presented in Table 2 and in panel C of Fig. S3–S5,† which represent the decay-associated difference spectra for each of the samples, respectively. For WT AR-3, short-lived components *τ*_1_ and *τ*_2_ are mainly attributed to the excited state decay and formation of the hot photoproduct. The decay time of the photoproduct (K intermediate) is beyond the detection time range of these experiments. For the mutants, on the other hand, *τ*_1_ is attributed to the thermal relaxation of the excited state and *τ*_2_ and *τ*_3_ to the excited state decay. Excited state lifetimes are consistent with the ones obtained in the FLUPS experiments. A direct comparison of the kinetic traces relevant to the excited state decay displays perfect agreement (not shown). The differences in the value of *τ*_1_ for WT AR-3 (Tables 1 and 2) are a consequence of the assumption of wavelength-independent lifetimes (global fit) for the TAS data.

**Table 2:** Global fitting results of TAS experiments

	WT AR-3	DETC	ARCH-5
*τ*_1_, ps	0.25 ± 0.02	0.4 ± 0.07	0.43 ± 0.03
*τ*_2_, ps	1.4 ± 0.2	7 ± 1	28 ± 5
*τ*_3_, ps	*∞*	42 ± 4	79 ± 6

### 2.4 Isomerization quantum yield

**Fig. 7:**
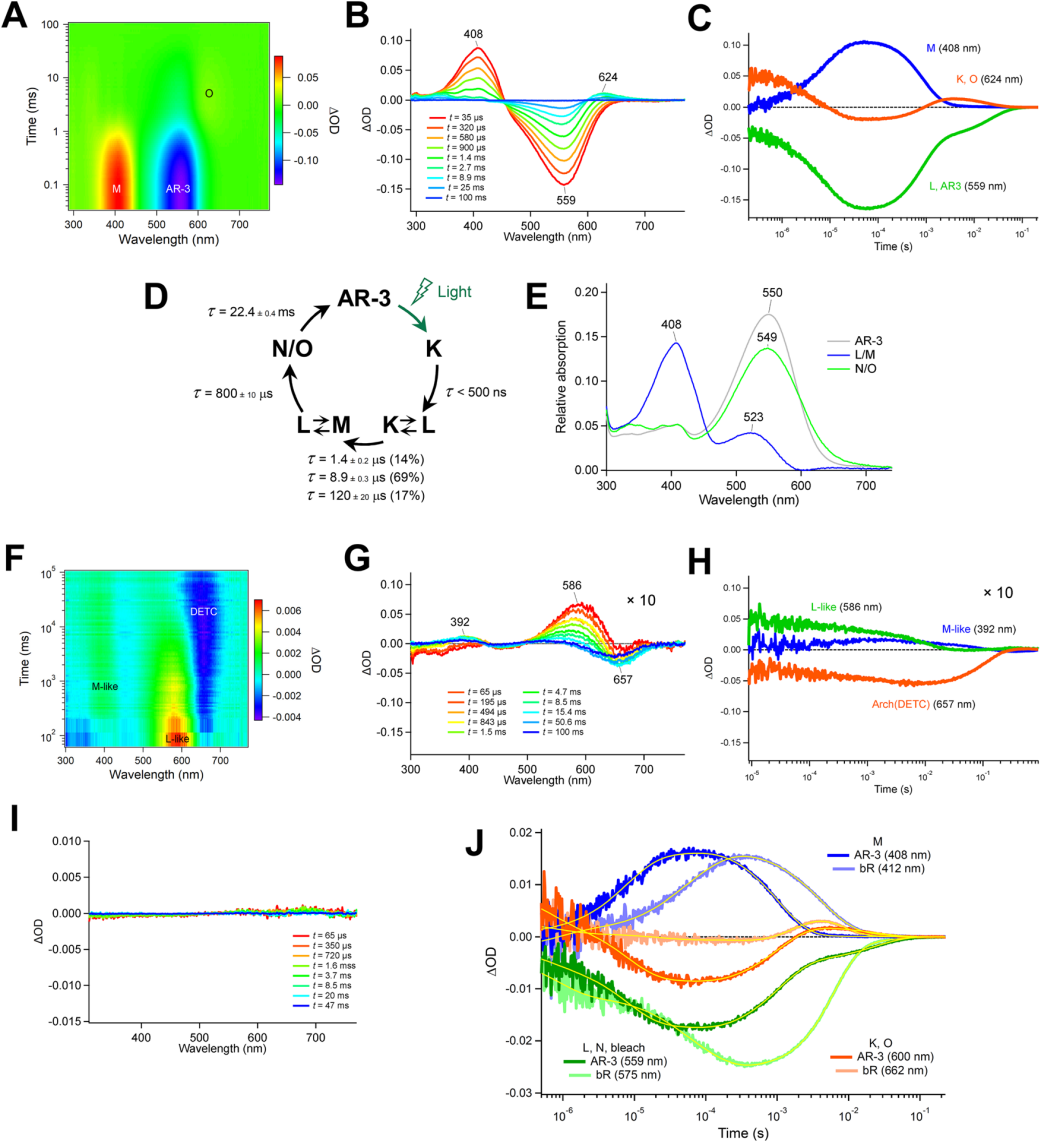
Transient absorption measurements of the LA WT AR-3, DETC, and ARCH-5 in the microsecond to millisecond time region. (A and F) 2D plot of the transient absorption change of the WT AR-3 (A), DETC (F). (B, G, and I) Transient absorption spectra of WT AR-3 (B), DETC (G), and ARCH-5 (I) at specific time delay (*t*) after the photoexcitation. (C) Time-evolutions of the transient absorption change of the WT AR-3 probed at 408, 559, and 624 nm representing the M, the L/AR-3, and the K/O, respectively. (D) The photocycle model of the WT AR-3 obtained by the multi-exponential analysis of the transient absorption change. (E) The calculated absorption spectra of the photo-intermediates. (H) Time-evolutions of the transient absorption change of DETC probed at 392, 586, and 657 nm representing the M-like, the L-like, and the DETC, respectively. (J) Transient absorption signals of the WT AR-3 and bR with 0.50 mJ cm^−2^ excitation pulse energy. For clarity, the signal intensities of G and H have been amplified tenfold.

To determine the isomerization quantum yield of the WT AR-3 (*Φ*^AR-3^_iso_), we performed a nano-second transient absorption measurement and compared the bleach signal intensity with bR. *Φ*^AR-3^_iso_is expressed as,^31^1
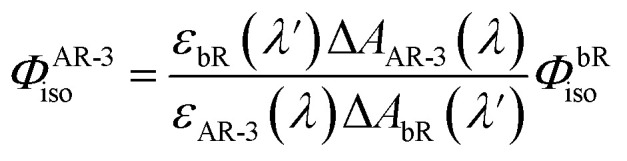
where *ε*_bR_(*λ*′) and Δ*A*_bR_(*λ*′) are the molecular extinction coefficient and the bleach signal of bR at probe wavelength *λ*′, *ε*_AR-3_(*λ*) and Δ*A*_AR-3_(*λ*) are the molecular extinction coefficient and the bleach signal of AR-3 at probe wavelength *λ*, respectively, and *Φ*^bR^_iso_ is the isomerization quantum yield of bR. The transient absorption spectra of light-adapted WT AR-3 were measured with 532 nm excitation wavelength (Fig. 7, panels A–C). At *t* = ∼100 μs, a blue-shifted M intermediate was accumulated. In this time region, the bleach signal of the initial state was observed without overlapping any absorption of red-shifted intermediates, *e.g.*, K and O intermediates. To extract the bleaching signal, the transient absorption change was analyzed by a multi-exponential function assuming an un-branched sequential reaction. To observe the short-lived photointermediates, a measurement at specific probe wavelengths with better time resolution using a photomultiplier tube was performed (Fig. 7, panel C). Based on the result of the multi-exponential analysis, we constructed a photocycle model of the WT AR-3 (Fig. 7, panel D) and calculated the absorption spectra of two photointermediates appearing after *t* = 35 μs. Comparing the order of appearance and peak wavelengths, they were assigned to the quasi-equilibrium between the L and the M and between the N and the O intermediates, (Fig. 7, panel E).^32^ Because there is a weak contribution of the L even at the time point where the M accumulation is maximized, we estimated the bleach signal at 600 nm probe wavelengths where no absorption of the photointermediate overlaps with the bleach signal at *t* = ∼100 μs. To compare the bleach signal intensity between AR-3 and bR, the samples were excited with low pulse energy (0.50 mJ cm^−2^) in the range where the linearity of the signal against the excitation pulse energy is maintained (Fig. S7, panel A†), and the absorptions of the WT AR-3 and bR sample solutions at the excitation wavelength were adjusted to the same with an error less than 1%. The transient absorption signals of the WT AR-3 and bR are shown in panel *J* of Fig. 7. Δ*A*_AR-3_(600 nm)/Δ*A*_bR_(575 nm) in eqn (1) was determined to be 0.36. To estimate the *ε*_AR-3_(*λ*), we observed the absorption change along with the hydrolysis reaction of the Schiff-base linkage of the retinal chromophore (Fig. S7, panel B†). The absorption of retinal oxime produced by the hydrolysis reaction was observed at 362 nm. Based on the difference molecular extinction coefficient of the retinal oxime (33 600 M^−1^ cm^−1^ (ref. 33)) and the ratio between the absorption of the retinal oxime and the bleaching of the initial AR-3, the *ε*_AR-3_ (600 nm) was determined to be 24 900 ± 300 M^−1^ cm^−1^ (*ε*_AR-3_ (559 nm) was 48 900 ± 300 M^−1^ cm^−1^ at the peak maximum). Based on these values, *Φ*^bR^_iso_ = 0.64 ± 0.04,^34^ and *ε*_bR_(575 nm) 62 000 ± 1000 M^−1^ cm^−1^,^35^*Φ*^AR-3^_iso_ was determined to be 0.54 ± 0.03.

Then, we investigated the photoreaction of DETC and ARCH-5 mutants. DETC excited at 600 nm exhibited two blue-shifted photointermediates at 586 and 392 nm (Fig. 7, panels F and G). Because the former appeared faster than the latter, whose absorption wavelength is in the near-UV region indicating to have a deprotonated retinal Schiff-base, the 586 and 392 nm species were named L-like and M-like intermediates. It was difficult to determine the precise lifetimes of these intermediates due to the extremely small transient absorption signals of DETC. The signal intensity of the transient absorption change of DETC is less than 1/20 of the WT AR-3, indicating its isomerization efficiency is considerably smaller than that of the WT AR-3, *i.e.* in the range of 0.02–0.03. ARCH-5 excited at 600 nm did not exhibit any significant transient absorption change (Fig. 7, panel I), indicating the photoisomerization reaction hardly occurs in this mutant.

We also searched for a photo-product signal on the sub-nanosecond time scale by performing femtosecond TAS. The K-like intermediate shows up in WT AR-3 as a pronounced absorption band peaking at 605 nm (Fig. 6A), red-shifted, as usual, with respect to the ground state absorption spectrum. Similar signatures hardly emerge from the noise for DETC and ARCH-5 in the range of 650–700 nm, and the very weak GSB is obscured by pump light scattering (570–600 nm, Fig. S6†). A quantitative evaluation of the quantum yield *Φ*_iso_ is therefore impossible, also due to the strong spectral overlap of GSB and the K-like absorption band. We note however, that these data are in agreement with the nano-second TAS (see above), in that ARCH-5 has a lower *Φ*_iso_ than DETC, and *Φ*^AR-3^_iso_ > 10 × *Φ*^DETC^_iso_.

The low *Φ*_iso_ values in the mutants support the conclusion that, relative to WT AR-3, the brighter steady-state emission of DETC and ARCH-5 at pH 6 is induced by a 1-photon absorption process, like in other AR-3-based GEVIs^22,23^ and in NeoR.^9^ Not only is the fluorescence intensity proportional to the excitation power, these mutants have a too low *Φ*_iso_ to form a putative fluorescent intermediate of sufficient amount, in stark contrast to WT AR-3. In addition, the fact that the ratio of the average fluorescence lifetimes agrees with the ratio of the *Φ*_f_'s for both mutants is in strong support of the 1-photon excitation process. Interestingly, a comparison with the S_1_ lifetime and *Φ*_f_ of NeoR^9^ shows that DETC and ARCH-5 have a radiative rate very similar to the former (*cf.*Table 1, *k*_r_ ≈ 1.7 × 10^8^ s^−1^ for NeoR). Note however that in both mutants, the RPSB is present in a mixture of all-*trans*/15-*anti* and 13-*cis*/15-*syn* isomers, as demonstrated by both the HPLC analysis and Raman spectroscopy. Light-adaptation only enhances the proportion of the all-*trans*/15-*anti*, but it remains limited to 30%. Both fluorescence up-conversion and TAS experiments show a bi-exponential excited state decay, with relative amplitudes similar to the isomer composition (*cf.*Table 1). The most direct interpretation is then that the decay times *τ*_1_ and *τ*_2_ represent the decay of all-*trans*/15-*anti* and 13-*cis*/15-*syn* isomers, respectively (Fig. S9†). This assignment may be questioned since for other microbial rhodopsins, it was shown that the excited state lifetime of 13-*cis*/15-*syn* is shorter than the one of all-*trans*/15-*anti*.^36–38^ The assignment is therefore preliminary and will be substantiated through ongoing work comparing the temperature-dependant excited state decays of both mutants in light- and dark-adapted states. Unfortunately, since the absorption spectra of both all-*trans*/15-*anti* and 13-*cis*/15-*syn* isomers largely overlap (Fig. S8B and C†), it is impossible to determine the *Φ*_iso_ and *Φ*_f_ independently for one or the other isomer. The quoted values are thus an average over the relative amount both forms.

## 3 Computational results and discussion

### 3.1 Computed excited state dynamics of AR-3

**Fig. 8:**
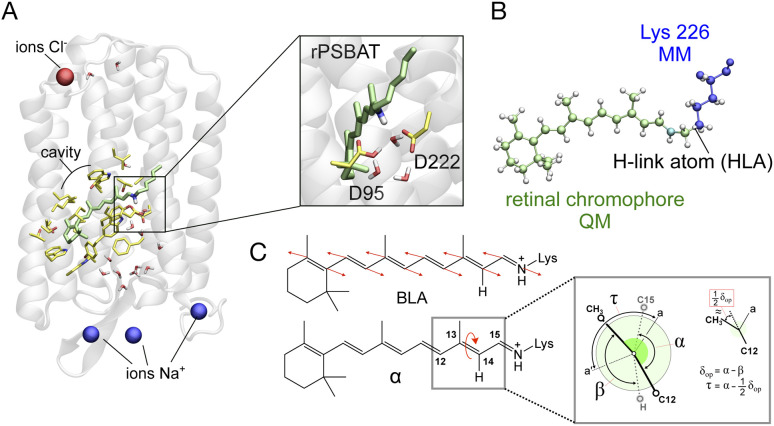
(A) Structure of the QM/MM model of AR-3. An inset shows a magnification of the RPSB region. (B) The QM/MM partitioning and description of the treatment of QM/MM frontier is exemplified. (C) Schematic representation of the geometrical coordinates *α*, *δ*_op_ and BLA dominating the *i*_1_ isomerization dynamics. The *τ* geometrical coordinate representing the orbital overlap across the π-bond is also defined.

We use an automatically constructed quantum mechanics molecular mechanics (QM/MM) model of AR-3 to simulate its room-temperature photoisomerization dynamics at the population level (see details in Fig. 8A, B and the Method section†). The result provides a validation of the constructed QM/MM model as well as a basis for the assignment of part of the spectroscopy data presented above. Most importantly, it discloses different mechanistic aspects of the photoisomerization dynamics in this class of archaea-rhodopsins at both the geometrical and electronic structure levels.

**Fig. 9:**
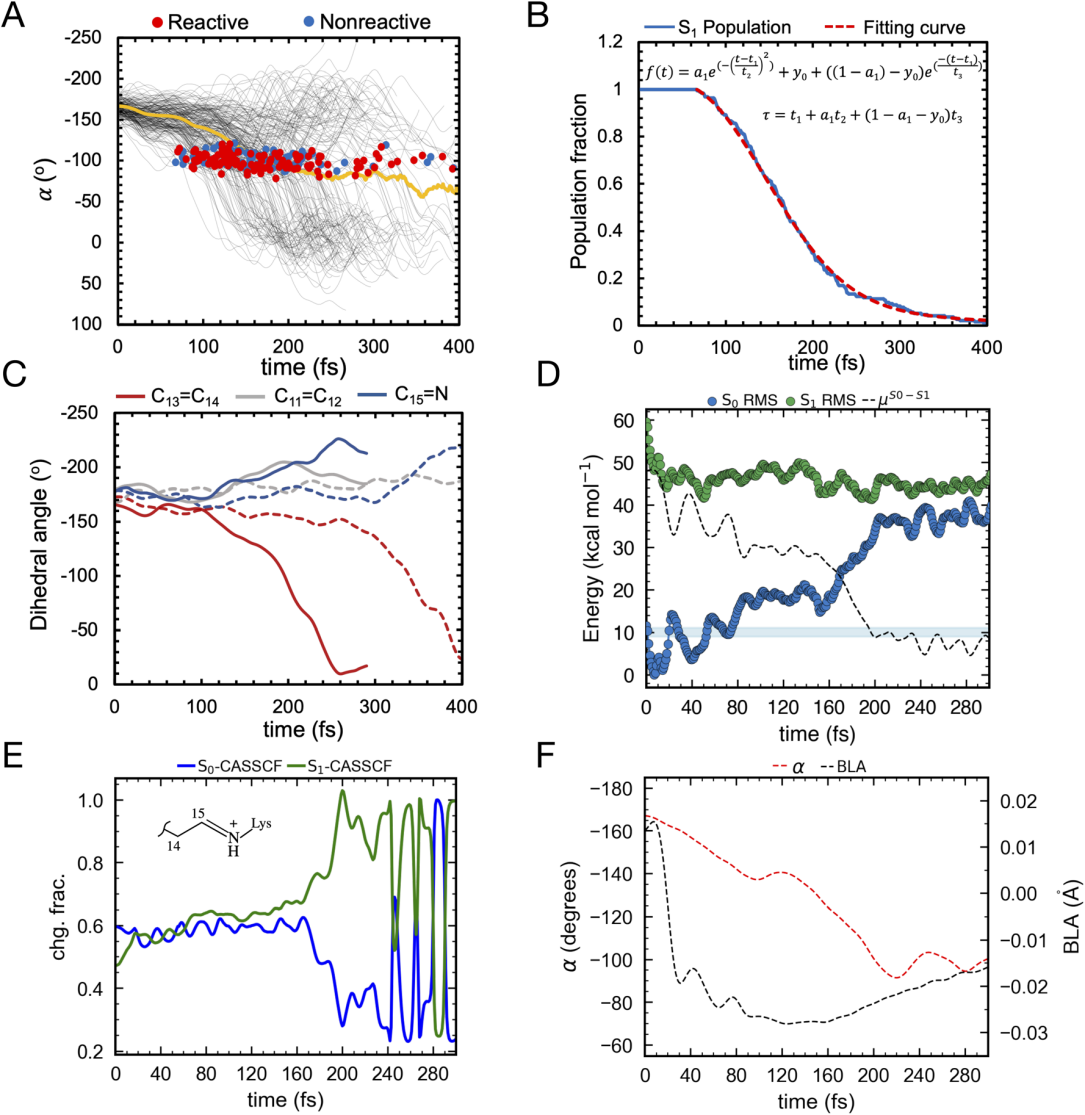
(A) Evolution of *α* in all-*trans* AR-3 WT population dynamics. The evolution of *α* along S_1_ trajectories (in black) and the corresponding average value (in yellow). Reactive (leading to isomerization) and non-reactive (aborted isomerization) decay points are represented by red and blue circles, respectively computed using 200 trajectories. (B) *τ* fitting from population dynamics (details in the ESI† and ref. 39). The S_1_ population fraction as a function of time and the fitting function are given in blue and red, respectively. (C) Change in average dihedral angles of C11=C12 (ash), C13=C14 (red), and C15=N (blue) bonds across five randomly selected fast (solid lines) and five slow (dashed lines) trajectories. (D) Energy profiles along a single RMS-CASPT2 FC trajectory for AR-3 WT assuming zero kinetic energy. S_0_ and S_1_ profiles are reported as blue and green diamonds, respectively. The average S_1_–S_0_ energy is also given, while the horizontal blue bar indicates a 10 kcal mol^−1^ threshold to emphasize the approaching of IS_S_1_/S_0__. (E) Evolution of the zeroth-order CASSCF charges associated to the AR-3 WT RMS-CASPT2 FC trajectory. The profiles correspond to the sum of such charges for the depicted moiety. (F) Evolution of *α* and BLA along the RMS-CASPT2 FC trajectory.

As illustrated in Fig. 9A the simulated isomerization, occurs on a sub-picosecond timescale indicating the existence of a substantially barrierless path connecting the Franck–Condon (FC) (*i.e.* vertical excitation) region of the population to an intersection seam (IS_S_1_/S_0__) formed by CoIn points with values of *α* (*i.e.* of the C12–C13–C14–C15 dihedral, see Fig. 8C) spanning a *ca.* 70–120° range. By fitting the change in the S_1_ population fraction as a function of simulation time (see Fig. 9B), it is possible to compute an *τ*_S_1__of ∼190 fs at the complete active space self-consistent field (CASSCF) level^39^(see ESI for details†). This should be compared with the average fluorescence lifetime result reported in Table 1 above, where a larger time constant of ∼340 fs is determined. In Fig. 9C we also show how the isomerization coordinate is dominated by the C13=C14 torsional deformation *α*.

While a ∼150 fs difference is significant when considering the short reaction time scale, we notice that this could, in part, depend on factors such as the limited accuracy of the PES and forces calculated at the CASSCF level that miss a significant portion of dynamic electron correlation energy. We will further discuss this point below. In Fig. 9A we also see that only part of the population reaches the photoproduct configuration (*i.e.* the K intermediate). By counting the number of reactive trajectories and calculating their fraction with respect to the total number of trajectories (reactive + unreactive), it is possible to estimate an *Φ*_iso_ that is 0.63 and, therefore, relatively close to the experimentally determined value of 0.54 ± 0.03. In conclusion, both the calculated *τ*_S_1__and *Φ*_iso_ seem to support a quantitatively correct use of the constructed AR-3 model for mechanistic studies.

An effort has been made to calculate a more accurate *τ*_S_1__value. To do so we used the state-of-the-art rotated multistate complete active space second-order perturbation theory (RMS-CASPT2)^40^ for equilibrium structures and trajectory calculation. In fact, like CASSCF, RMS-CASPT2 correctly describes the intersecting S_1_ and S_0_ PESs near the CoIn but also accounts for the dynamic electron correlation missing in CASSCF energy and gradient computations.^40,41^ In spite of their availability, running hundreds of RMS-CASPT2 quantum-classical trajectories is still highly unpractical. Therefore, we determining a scaling coefficient “*a*_scale_”, relating CASSCF and RMS-CASPT2 gradients and resulting in a correction for the *τ*_S_1__(*t*^RMS-CASPT2^ = *a*_scale_ × *t*^CASSCF^, details in the ESI† and ref. 42). The resulting *a*_scale_ value of 1.25 produces an improved *τ*_S_1__ of 239 fs. This finding suggests that, as expected from previous studies^43^ the inclusion of dynamic electron correlation leads to a flatter S_1_ PES, or on the contrary, that the CASSCF method tends to produce systematically steeper excited state PESs.

To confirm the conclusion above, we were able to propagate a single RMS-CASPT2 quantum-classical trajectory released from the FC geometry with zero initial kinetic energy to approximately estimate the average dynamics of the population.^44^ As shown in Fig. 9D, we found that such a trajectory indeed spans a shallower PES. The time necessary to approach the IS_S_1_/S_0__, that we conveniently define with a threshold of *e.g.* 10 kcal mol^−1^ (see Fig. 9D) is significantly longer than in the corresponding CASSCF trajectory consistent with the scaling. However, a significant discrepancy between the computationally corrected *τ*_S_1__ and the experimental one remains, which is most probably due to our computations being based on a zero initial velocity single FC trajectory which does not properly account for the real ensemble of populations at room temperature.

### 3.2 Mechanistic aspects

In this section we look at some characteristics of the FC trajectory computed at the RMS-CASPT2 (and for comparison CASSCF) level of theory to discuss the general structural and electronic features of the S_1_ relaxation of AR-3 towards the IS_S_1_/S_0__. We will then use the population dynamics simulated at the CASSCF level to discuss additional mechanistic features emerging from the population statistics. As shown in Fig. 9E, in the FC geometry the S_0_ state of WT AR-3 is characterized by a mixed COV/CT electronic character, *i.e.* its wavefunction is described in terms of these “diabatic states” (see also next section). In more detail, the S_0_ delocalized iminium positive charge is primarily (60%) residing in the chromophore portion incorporating the Schiff base moiety (*e.g.* with respect to the C13=C14 bond, see inset) and in S_1_ away from the Schiff base moiety. This situation changes rapidly, after 160 fs, along the S_1_ relaxation where the S_0_ state becomes dominated by the CT character and the S_1_ state by the COV character.

This rather distinct behavior with respect to, for instance, animal rhodopins^45^ has been previously reported for certain AR-3 variants^11^ but, also, for extremely red-shifted rhodopsins such as NeoR.^46^ As the C13=C14 bond twists, S_1_ further increases in COV character until an *α* value of ≈90° (see Fig. 9F) where the π-conjugation along the chromophore backbone is substantially broken and now S_1_ features an almost pure COV character. The S_1_ electronic character change is mirrored by less negative values of the BLA. In fact, as displayed in Fig. 9F, the BLA value (*i.e.* the difference between average single and average double bond lengths) increases as the COV character increases, along the trajectory.

As noted in a previous contribution by some of the authors,^11,46^ as the conjugation is broken, the COV character coincides with that of two unpaired electrons, thus it is more appropriate to reference this area of the S_1_ PES as that of a short lived (or even transient) TIDIR as, indeed, a shallow S_1_ energy minimum could be located. The COV character appears evident both by looking at the charges of the zeroth-order CASSCF and RMS-CASPT2 wavefunctions. In fact, this is naturally related to non-dynamic rather than dynamic electron correlation. Further inspection of Fig. 9D and F shows, consistently with existence of a TIDIR species, a small trajectory segment where the S_1_ energy, S_1_–S_0_ energy gap and COV character do not change. In the case of the CASSCF wavefunction the nature of the S_1_ electronic character oscillates revealing the presence of a nearby CoIn that, presumably, is not crossed in the RMS-CASPT2 description.

Going back to the CASSCF/6-31G* population dynamics, inspection of the geometrical changes associated with other relevant torsional degrees of freedom and accompanying the progression of *α* (see Fig. 8C) along the S_1_ PES are documented in Fig. 9C. One can see that both the C15=N and C11=C12 bonds (measured using the skeletal dihedral angles C14–C15–N–C and C10–C11–C12–C13, respectively) undergo significant twisting in the counterclockwise direction relative to the clockwise *α* change and, thus, revealing a bicycle pedal motion active at the population level irrespective of whether the decay is slow or fast. On the other hand, such coupled motion is aborted upon decay as the “reactive” trajectories produce exclusively the 13-*cis* isomer of the chromophore upon S_0_ relaxation. This is reminiscent of the isomerization coordinate of other animal^47^ and microbial rhodopsins^48,49^ as originally proposed by Warshel.^47^

**Fig. 10:**
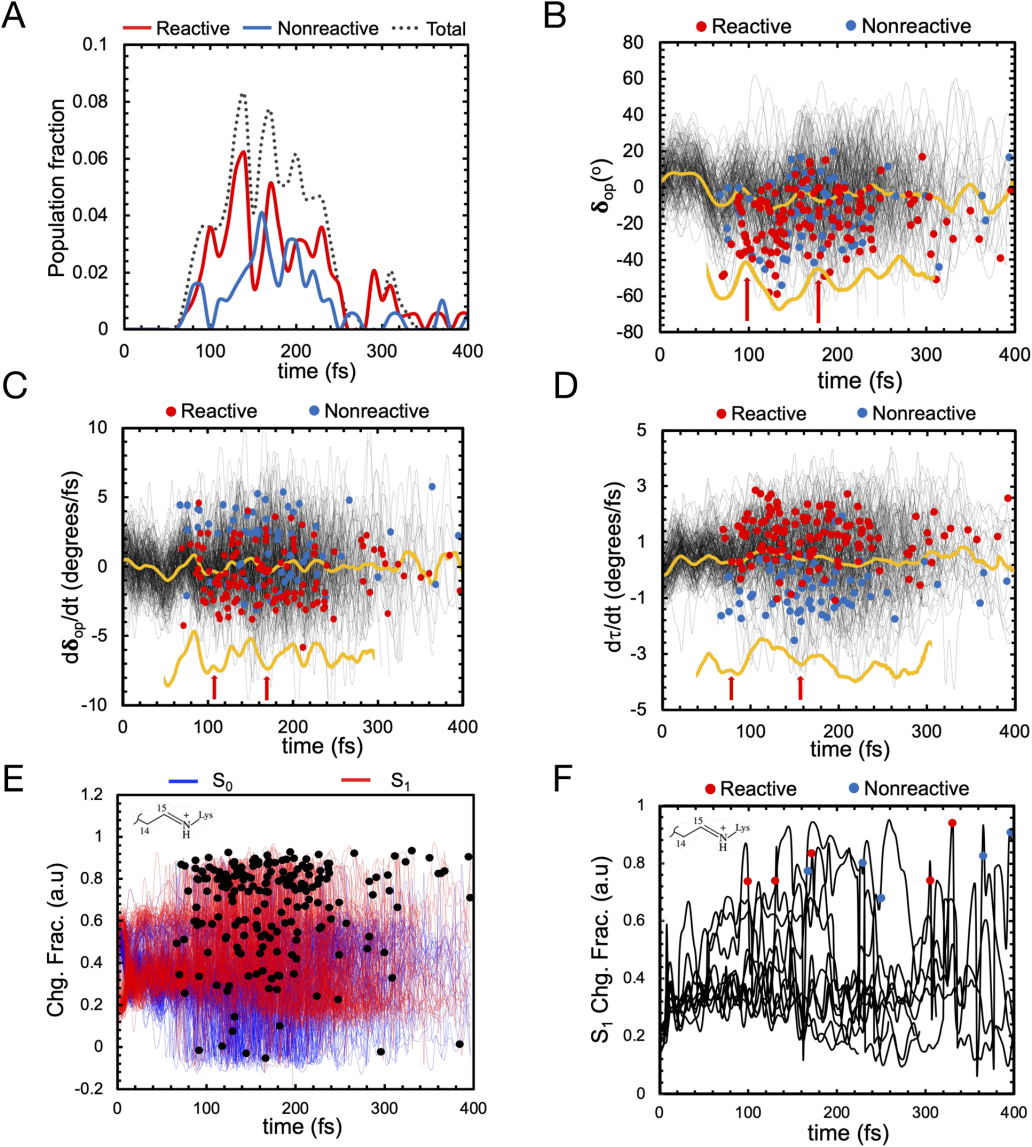
(A) Oscillatory character of the S_1_ total population decay (dotted line), reactive population decay (red) and non-reactive population decay (blue). (B) *δ*_op_ progression of S_1_ population (in black) and corresponding average value (in yellow). Reactive (leading to isomerization) and non-reactive (aborted isomerization) decay points are represented by red and blue circles, respectively in figures B, C, D and F computed using 200 trajectories (C) *δ*_op_ progression velocity of S_1_ population (black) and corresponding average value (yellow). (D) *τ* progression velocity of S_1_ population (in black) and corresponding average value (in yellow). (E) Evolution of the zeroth-order CASSCF charges on the Schiff base moiety associated with S_0_ (blue) and S_1_ state (red). S_1_ decay points represented in black dots. (F) Evolution of the zeroth-order CASSCF charge associated with S_1_ state developed using ten randomly selected trajectories for the same moiety.

Let us now discuss the vibrationally coherent character of the excited state reaction. Inspection of the distribution of the reactive “hop” events (see red line, Fig. 10A) displays, for delays of 100–250 fs, at least two reactive waves that are separated by a *ca.* 40–50 fs period. Importantly, a plot of the geometrical variable *δ*_op_ (see Fig. 10B) corresponding to an out-of-plane hydrogen wag at C14 somehow coupled with the wag of the heavier methyl substituent at C13, displays a similar periodicity indicating an impact of the *δ*_op_ wag phase at the time of the decay on the trajectory reactivity. Indeed, these data support the hypothesis that the so-called “HOOP mechanism” demonstrated in bovine rhodopsin is also active in this microbial rhodopsin whose isomerization occurs on the C13=C14 double bond. Thus the AR-3 simulation provides evidence that the replacement of a H–C11=C12–H moiety of animal rhodopsins with the Me–C13=C14–H moiety of microbial rhodopsins does not quench the HOOP mechanism for the control of trajectory reactivity (and, in turn, *Φ*_iso_, see ref. 45) through a phase matching between the Me–C13=C14–H wag velocity and the *α* velocity. This implies that the larger is the population fraction decaying with a negative *δ*_op_ velocity at decay (the phase of *α* at decay is statistically positive due to the clockwise motion), the higher is the achieved *Φ*_iso_ value. Similar to the case of H–C11=C12–H wag in animal rhodopsins, the phase dependent reactivity is explained by a change in overlap velocity at the moment of decay.^45^ This is illustrated by the compound geometrical variable *τ* (see Fig. 8C above), which is a combination of the variable describing the Me–C13=C14–H wag (HOOP) and the variable describing the C13=C14 isomerization (*α*), and that is proportional to the overlap between the fragment of π-orbitals whose interaction generates the C13=C14 π bond. The figure shows how the phase of the *τ* velocity is substantially dominated by the phase of the *δ*_op_ velocity (Fig. 10C) and that a positive *τ* velocity at decay marks reactive trajectories (see Fig. 10D).

The above results convey a wealth of new information both on the S_1_ dynamics as well as on the optogenetically relevance of WT AR-3 as a template for the development of novel fluorescent reporters (see next section). In fact, on one hand, the spectroscopy of LA WT AR-3 at pH 6, shows that both the fluorescence decay kinetics and the ultrafast isomerization reaction monitored by TAS are consistent with several examples reported in the literature for other microbial rhodopsins, including bR.^50–54^ The dominant ≈300 fs decay time is due to the all-*trans*-to-13-*cis* isomerization process; an assignment supported by the QM/MM-based simulation of the light-induced population dynamics. In addition, these calculations also reveal that photoisomerisation occurs *via* a bicycle-pedal mechanism similar to the one found in animal rhodopsins (but occurring on the C13=C14–C15=N moiety rather than the C9=C10–C11=C12) and that *Φ*_iso_ is modulated by coherent oscillations of Me–C13=C14–H wagging motion, which is a feature of barrier-less and ballistic isomerisation as seen, again, in animal rhodopins.^45^

**Fig. 11:**
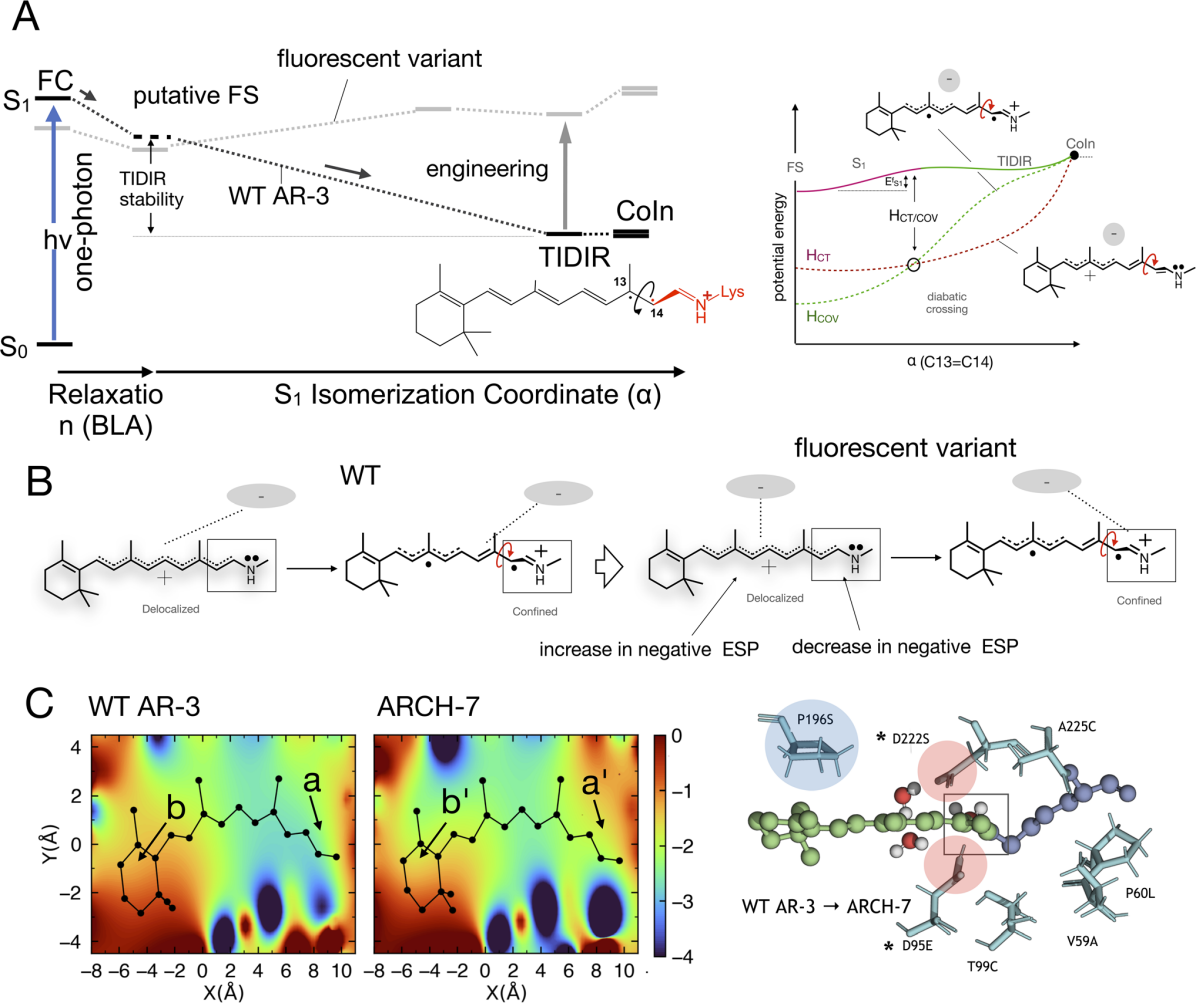
(A) Schematic comparison between the WT AR-3 S_1_ isomerization energy profiles (black) and the energy profile of a strongly emissive AR-3 variant (grey) (see also Fig. 1). It is evident that an engineering effort targeting high fluorescent AR-3 variants must destabilize the TIDIR region relative to the putative fluorescent state FS (bold vertical arrow). (B) Representation of the ESP changes through the displacement of a negative charge centroid when shifting from WT AR-3 to a fluorescent variant. (C) ESP cross-section computed for a WT AR-3 model and for the model of its ARCH-7 variant. A representation of the chromophore cavity mutation sites affecting the ESP is shown on the right (panel adapted from a previous work).^11^ The position of two relevant carboxylic side chains is marked with a*.

On the other hand, and in spite of the above similarities, it is shown here for the first time that WT AR-3 is electronically different from reference (*e.g.* bR) microbial rhodopsins and animal rhodopsins: it has an unusual degree of mixed COV and CT characters (described by the chromophore positive charge distribution. See Fig. 9E, 10E and F) in both the S_0_ and S_1_ states already present in the Franck–Condon region and then conserved all along the reaction path. This and the related existence of a TIDIR intermediate close to the CoIn support the hypothesis that certain (presently unknown) fluorescent mutants of AR-3 may behave like NeoR.^11,46^ However, WT AR-3 shows that the conclusion indicating that the an S_1_ TIDIR with COV character necessarily implies an isomerisation-preventing S_1_ barrier^20^ is not valid any more. It is rather the destabilization of a pre-existing TIDIR “transient state” that modulates the barrier. It remains to be understood which exact electrostatic features of the RPSB-binding protein pocket WT AR-3, its mutants and in NeoR lead to their unusual electronic properties, *i.e.* the formation of the S_1_ TIDIR with COV character (*cf.*Fig. 11).

## 4 Engineering aspects

When considering the rational engineering of AR-3 towards more fluorescent variants, one has to deal with the overwhelming structural complexity of the cavity hosting the chromophore. Indeed, the fact that the cavity may incorporate the chromophore counterion in different positions and polar residues in different orientations in response to both electrostatic and steric factors, leads to patterns of electrostatic potential (ESP) and steric interactions whose effect is not obvious and very difficult to predict. For this reason, here we do not explicitly focus on the effect of amino acid replacements but, rather, on the changes in cavity electrostatics that, in principle, would lead to an increased excited state lifetime of DETC and ARCH-5 and other fluorescent AR-3 variants. This is assumed to be the most important factor for controlling the 1-photon *Φ*_f_.

We start by noticing that the AR-3 population dynamics reported above shows that the S_1_ electronic character changes accompanying the isomerization, are confirmed at the statistical level. Indeed, Fig. 10E and F show that the majority of trajectories display a COV character at the moment of S_1_ decay (S_1_ Schiff base charge fraction >0.8). In other words, the theory of photoisomerization in AR-3 mutants and NeoR^11,46^ illustrated in Fig. 1 must also apply to WT AR-3. Again, this is different from the classical picture documented for bovine rhodopsin^45^ and bacteriorhodopsin,^55^ according to which S_1_ has a CT character at decay (S_1_ Schiff base charge fraction <0.2). It is this specific electronic character of the AR-3 chromophore that accounts for the impact of site-specific mutations on the variants' S_1_ lifetime.^14–16,44^

We now discuss which exact electrostatic features of the RPSB-binding protein pocket of WT AR-3 have to change to generate its brighter mutants such as DETC and ARCH-5 or, in perspective, variants as bright as NeoR. As anticipated above, our reasoning relies on the formation of the S_1_ TIDIR with COV character (*cf.* left panel in Fig. 11A). This implies that, (i) along the C13=C14 isomerization coordinate the diabatic states representing the CT and COV electronic characters cross (*cf.* right panel in Fig. 11A)^11^ and that the height of the crossing is proportional to the 
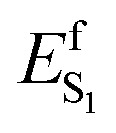
 barrier, (ii) the cavity electrostatics acts according to a mechanism called “charge delocalization and confinement”^46^ illustrated in Fig. 11B, and (iii) due to the initial mixed CT/COV character the FC and putative FS regions feature a chromophore with a “delocalized” positive charge while such charge is firmly “confined” in the C–C=N moiety in TIDIR (or the CoIn region accessed by the trajectories). The consequence is that an increase of negative ESP (or a decrease of positive ESP) in a chromophore cavity region located far from the C–C=N moiety must destabilize the TIDIR region and increase 
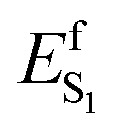
 (see Fig. 11A and B). Due to lack of structural data, we could not computationally assess the above hypothesis for DETC and ARCH-5. Thus, in Fig. 11C we compare the ESP cross-section of WT AR-3 and its bright variant ARCH-7 whose QM/MM model has been reported.^11^ It can be seen that the electrostatic potential becomes more positive in the C–C=N moiety (compare a with a′) region and more negative in the region close to the chromophore ring (compare b and b′) when going from WT to the variant.

Of course, these changes in ESP must be induced by the corresponding amino acid replacements that are shown on the right-end of the same figure. For instance, the replacements of the negatively charged oxygens of residue 222 with a single neutral serine oxygen combined with transferring the lost negative charge to residue 95 at a larger distance from the Schiff base, would contribute to projecting a less negative ESP on the C–C=N moiety. Similarly, the proline to serine replacement at position 196 could contribute to increasing the negative ESP far from the Schiff base. However, while one can attempt a rationalization of the ESP changes on the basis of the specific change in partial charge distribution and orientation induced by the replacements, the “inverse” problem of predicting which amino acid replacements would induce the wanted cavity ESP appears extremely complex and presently difficult to tackle if not in a highly hypothetical fashion. For this reason, we do not further elaborate on such point.

Regarding the voltage sensitivity of the fluorescence intensity, in AR-3, its D95N mutant, and Archon1, its origin is proposed to result from the voltage-dependent change in the hydrogen-bonding network. These changes are induced by shifts in the orientation of R92 or D125, which in turn affect the equilibrium between the protonated and deprotonated states of the RSB, leading to the change in the fluorescent state.^1,22,56^ Therefore, to improve the fluorescence quantum yields of AR-3 variants while maintaining their voltage sensitivity, amino acid residues not involved in the hydrogen bonding network around RSB, R82, and D125 should be targeted for mutation to increase the excited state barrier.

## 5 Conclusions

In the context of establishing WT AR-3 as an ideal template for engineering fluorescent GEVIs, we motivated the present combined experimental and computational study by two questions: does the TIDIR intermediate with COV character also exist in WT AR-3, and how to provide experimental evidence for the TIDIR-related PES barrier in mutants? The latter is addressed by a set of spectroscopic studies of the DETC and ARCH-5 mutants, which highlight the extended, barrier-induced excited state lifetimes of the all-*trans*/15-*anti* and 13-*cis*/15-*syn* isomers; a fact also found in other AR-3-based mutants.^22,23^ In addition, the larger excited state barriers in the mutants correlate with a largely reduced isomerization quantum yield, *Φ*_iso_. The first question is addressed by an unprecedented non-adiabatic dynamics simulation of the WT AR-3 photoisomerization at the population level, which allows to compute both the excited state lifetime and *Φ*_iso_ thus providing a basis for a mechanistic interpretation of the experimental results. The most important message from the simulated population dynamics is that the WT AR-3 displays indeed a significant CT character in S_0_ and a TIDIR intermediate with COV character in S_1_. This supports the hypothesis that the WT form of AR-3 has the same type of electronic character as the one found computationally in its weakly fluorescent AR-3 variants as well as in the strongly fluorescent NeoR. However, WT AR-3 does not feature an S_1_ isomerization barrier (
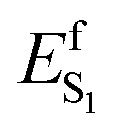
 is negligible or absent), and therefore decays rapidly to S_0_. Since other microbial rhodopsins failed to produce fluorescent variants with *Φ*_f_'s similar to the ones obtained with AR-3 variants,^15^ we conjecture that the specific COV/CT mixture in S_0_ and S_1_, as well as the presence of a TIDIR intermediate with full COV character, is the reason for the WT AR-3 propensity to form isomerisation-restraining S_1_ barriers upon suitable genetic engineering.

The mechanism for the transition from WT AR-3 to a fluorescent form has been discussed and justified under the hypothesis that is controlled by variations in the cavity electrostatics and its interaction with the delocalized positive charge computed near the FC point and the TIDIR charge that is confined in the Schiff base moiety. This provides a rational route to engineering. In fact, it is possible to conclude that the problem of engineering highly fluorescent AR-3 variants, namely to increase the *τ*_S_1__of the system, can be solved by finding mutations creating an ESP that destabilizes TIDIR with respect to FS. However, the problem of how to exactly identify the necessary mutation pattern (or group of different mutation patterns) remains to be further investigated.

## Data availability

Additional data for this article, including HPLC, time-resolved spectroscopy, computational results, and the calculation of average fluorescence lifetimes are available in the ESI.†

## Author contributions

The present paper is the result of a collaborative project of the teams led by M. O., M. S., K. I. and S. H. M. K. expressed and purified the proteins. K. H., M. K., I. W. and M. S. carried out the experiments and collected the data. D. W. and L. B. generated the QM/MM models and carried out all the calculations. All the authors contributed to analyzing, discussing, and interpreting the results, and to improving the different versions of the manuscript. K. H., D. W., M. K., L. B., M. O., K. I. and S. H. wrote the manuscript and ESI.†

## Conflicts of interest

The authors declare no conflict of interest.

## Supplementary Material

SC-OLF-D4SC05120C-s001

## References

[cit1] Meng X., Ganapathy S., van Roemburg L., Post M., Brinks D. (2023). ACS Phys. Chem. Au.

[cit2] Kandori H. (2020). Bull. Chem. Soc. Jpn..

[cit3] Kralj J., Douglass A., Hochbaum D. (2012). Nat. Methods.

[cit4] Kojima K., Shibukawa A., Sudo Y. (2020). Biochemistry.

[cit5] McIsaac R. S., Engqvist M. K. M., Wannier T., Rosenthal A. Z., Herwiga L., Flytzanis N. C., Imashevac E. S., Lanyic J. K., Balashovc S. P., Gradinarub V., Arnolda F. H. (2014). Proc. Natl. Acad. Sci. U.S.A..

[cit6] Flytzanis N. C., Bedbrook C. N., Chiu H., Engqvist M. K. M., Xiao C., Chan K. Y., Sternberg P. W., Arnold F. H., Gradinaru V. (2014). Nat. Commun..

[cit7] Hochbaum D. R., Zhao Y., Farhi S. L., Klapoetke N., Christopher A Werley V. K., Zou P., Kralj J. M., Dougal Maclaurin N. S.-M., Saulnier J. L., Boulting G. L., Straub C., Cho Y. K., Melkonian M., Wong G. K.-S., Harrison D. J., Murthy V. N., Sabatini B. L., Boyden E. S., Campbell R. E., Cohen A. E. (2014). Nat. Methods.

[cit8] Piatkevich K. D., Jung E. E., Straub C., Linghu C., Park D., Suk H.-J., Hochbaum D. R., Goodwin D., Pnevmatikakis E., Pak N., Kawashima T., Yang C.-T., Rhoades J. L., Shemesh O., Asano S., Yoon Y.-G., Freifeld L., Saulnier J. L., Riegler C., Engert F., Hughes T., Drobizhev M., Szabo B., Ahrens M. B., Flavell S. W., Sabatini B. L., Boyden E. S. (2018). Nat. Chem. Biol..

[cit9] Broser M., Spreen A., Konold P. E., Peter E., Adam S., Borin V., Schapiro I., Seifert R., Kennis J. T. M., Bernal Sierra Y. A., Hegemann P. (2020). Nat. Commun..

[cit10] Palombo R., Barneschi L., Pedraza-González L., Padula D., Schapiro I., Olivucci M. (2022). Nat. Commun..

[cit11] Barneschi L., Marsili E., Pedraza-González L., Padula D., De Vico L., Kaliakin D., Blanco-González A., Ferré N., Huix-Rotllant M., Filatov M., Olivucci M. (2022). Nat. Commun..

[cit12] Maclaurin D., Venkatachalam V., Lee H., Cohen A. E. (2013). Proc. Natl. Acad. Sci. U.S.A..

[cit13] Kojima K., Kurihara R., Sakamoto M., Takanashi T., Kuramochi H., Zhang X. M., Bito H., Tahara T., Sudo Y. (2020). J. Phys. Chem. B.

[cit14] Gozem S., Luk H. L., Schapiro I., Olivucci M. (2017). Chem. Rev..

[cit15] Marín M. d. C., Agathangelou D., Orozco-Gonzalez Y., Valentini A., Kato Y., Abe-Yoshizumi R., Kandori H., Choi A., Jung K.-H., Haacke S., Olivucci M. (2019). J. Am. Chem. Soc..

[cit16] Agathangelou D., Roy P. P., del Carmen Marín M., Ferré N., Olivucci M., Buckup T., Léonard J., Haacke S. (2021). C. R. Phys..

[cit17] Zhang J., Singh P., Engel D., Fingerhut B. P., Broser M., Hegemann P., Elsaesser T. (2024). Proc. Natl. Acad. Sci. U.S.A..

[cit18] Schenkl S., van Mourik F., van der Zwan G., Haacke S., Chergui M. (2005). Science.

[cit19] Léonard J., Portuondo-Campa E., Cannizzo A., van Mourik F., van der Zwan G., Tittor J., Haacke S., Chergui M. (2009). Proc. Natl. Acad. Sci. U.S.A..

[cit20] Broser M., Andruniów T., Kraskov A., Palombo R., Katz S., Kloz M., Dostál J., Bernardo C., Kennis J. T. M., Hegemann P., Olivucci M., Hildebrandt P. (2023). J. Phys. Chem. Lett..

[cit21] Penzkofer A., Silapetere A., Hegemann P. (2019). Int. J. Mol. Sci..

[cit22] Silapetere A., Hwang S., Hontani Y., Fernandez Lahore R. G., Balke J., Velazquez Escobar F., Tros M., Konold P. E., Matis R., Croce R., Walla P. J., Hildebrandt P., Alexiev U., Kennis J. T. M., Sun H., Utesch T., Hegemann P. (2022). Nat. Commun..

[cit23] Nikolaev D. M., Mironov V. N., Metelkina E. M., Shtyrov A. A., Mereshchenko A. S., Demidov N. A., Vyazmin S. Y., Tennikova T. B., Moskalenko S. E., Bondarev S. A., Zhouravleva G. A., Vasin A. V., Panov M. S., Ryazantsev M. N. (2024). ACS Phys. Chem. Au.

[cit24] Oesterhelt S., Meentzen M., Schuhmann L. (1973). Eur. J. Biochem..

[cit25] Smith S. O., Braiman M. S., Myers A. B., Pardoen J. A., Courtin J. M. L., Winkel C., Lugtenburg J., Mathies R. A. (1987). J. Am. Chem. Soc..

[cit26] Smith S. O., Pardoen J. A., Lugtenburg J., Mathies R. A. (1987). J. Phys. Chem..

[cit27] Léonard J. , Gelot T., Torgasin K. and Haacke S., 9th International Conference on Photonics and Imaging in Biology and Medicine (Pibm 2010), 2011, vol. 277

[cit28] Gerecke M., Bierhance G., Gutmann M., Ernsting N. P., Rosspeintner A. (2016). Rev. Sci. Instrum..

[cit29] Hasson K., Gai F., Anfinrud P. A. (1996). Proc. Natl. Acad. Sci. U.S.A..

[cit30] Bismuth O., Friedman N., Sheves M., Ruhman S. (2007). J. Phys. Chem. B.

[cit31] Inoue K., Sudo Y., Homma M., Kandori H. (2011). J. Phys. Chem. B.

[cit32] Chizhov I., Chernavskii D. S., Engelhard M., Mueller K. H., Zubov B. V., Hess B. (1996). Biophys. J..

[cit33] Scharf B., Pevec B., Hess B., Engelhard M. (1992). Eur. J. Biochem..

[cit34] Tittor J., Oesterhelt D. (1990). FEBS Lett..

[cit35] Hashimoto S., Obata K., Takeuchi H., Needleman R., Lanyi J. K. (1997). Biochemistry.

[cit36] Wand A., Rozin R., Eliash T., Jung K.-H., Sheves M., Ruhman S. (2011). J. Am. Chem. Soc..

[cit37] Wand A., Friedman N., Sheves M., Ruhman S. (2012). J. Phys. Chem. B.

[cit38] Cheminal A., Léonard J., Kim S.-Y., Jung K.-H., Kandori H., Haacke S. (2015). Phys. Chem. Chem. Phys..

[cit39] Manathunga M., Yang X., Luk H. L., Gozem S., Frutos L. M., Valentini A., Ferrè N., Olivucci M. (2016). J. Chem. Theory Comput..

[cit40] Battaglia S., Lindh R. (2020). J. Chem. Theory Comput..

[cit41] Nishimoto Y., Battaglia S., Lindh R. (2022). J. Chem. Theory Comput..

[cit42] Frutos L. M., Andruniów T., Santoro F., Ferré N., Olivucci M. (2007). Proc. Natl. Acad. Sci. U.S.A..

[cit43] Barneschi L., Kaliakin D., Huix-Rotllant M., Ferré N., Filatov M., Olivucci M. (2023). J. Chem. Theory Comput..

[cit44] Gozem S., Luk H. L., Schapiro I., Olivucci M. (2017). Chem. Rev..

[cit45] Yang X., Manathunga M., Gozem S., Léonard J., Andruniów T., Olivucci M. (2022). Nat. Chem..

[cit46] Palombo R., Barneschi L., Pedraza-González L., Padula D., Schapiro I., Olivucci M. (2022). Nat. Commun..

[cit47] Warshel A. (1976). Nature.

[cit48] Palombo R., Barneschi L., Pedraza-González L., Yang X., Olivucci M. (2024). Phys. Chem. Chem. Phys..

[cit49] Altoè P., Cembran A., Olivucci M., Garavelli M. (2010). Proc. Natl. Acad. Sci. U.S.A..

[cit50] Mathies R. A., Brito Cruz C. H., Pollard W. T., Shank C. V. (1988). Science.

[cit51] Du M., Fleming G. R. (1993). Biophys. Chem..

[cit52] Hasson K., Gai F., Anfinrud P. (1996). Proc. Natl. Acad. Sci. U.S.A..

[cit53] Briand J., Léonard J., Haacke S. (2010). J. Opt..

[cit54] Wand A., Gdor I., Zhu J., Sheves M., Ruhman S. (2013). Annu. Rev. Phys. Chem..

[cit55] Gozem S., Johnson P. J., Halpin A., Luk H. L., Morizumi T., Prokhorenko V. I., Ernst O. P., Olivucci M., Miller R. D. (2020). J. Phys. Chem. Lett..

[cit56] Saint Clair E. C., Ogren J. I., Mamaev S., Russano D., Kralj J. M., Rothschild K. J. (2012). J. Phys. Chem. B.

