# Supplementary material for: Archaerhodopsin 3 is an ideal template for the engineering of highly fluorescent optogenetic reporters
Source: Chem Sci. 2024 Nov 18;16(2):761–74. doi: 10.1039/d4sc05120c (PMC11612921; doi:10.1039/d4sc05120c)
Supplement: SC-016-D4SC05120C-s001 [file SC-016-D4SC05120C-s001.pdf]

Supplementary Information (SI) for Chemical Science.
This journal is © The Royal Society of Chemistry 2024

Archaerhodopsin 3 is an Ideal Template for the Engineering of Highly Fluorescent Optogenetic Reporters

November 15, 2024

Krystyna Herasymenko[a], Danushka Walisinghe[b], Masae Kono[c], Leonardo Barneschi[d], Isabelle de Waele[e], Michel Sliwa[e,f], Keiichi Inoue[c], Massimo Olivucci[b,d] and Stefan Haacke[a]

Contents

1 Methods **1**
1.1 VIS-NIR femtosecond transient absorption spectroscopy . . . 1
1.2 Fluorescence up-conversion spectroscopy . . . 2
1.3 Raman spectroscopy . . . 2
1.4 Fluorescence spectroscopy . . . 2
1.5 UV-VIS Absorption spectroscopy . . . 2
1.6 Protein expression in *Escherichia coli* cells . . . 2
1.7 Protein purification . . . 3
1.8 Retinal configuration analysis by HPLC . . . 3
1.9 UV-VIS nanosecond transient absorption spectroscopy . . . 3
1.10 QM/MM model construction and validation . . . 4
1.11 Non-adiabatic trajectory computation . . . 4

2 Supplementary data **6**
2.1 Fluorescence up-conversion spectroscopy, FLUPS . . . 6
2.2 Average fluorescence lifetimes and quantum yield . . . 7
2.3 Transient Absorption Spectroscopy . . . 8
2.4 UV-VIS nanosecond transient absorption spectroscopy . . . 12
2.5 Light adaptation effect . . . 13
2.5.1 Protocol and effects of light adaptation on the steady-state absorption spectra . . . 13
2.5.2 Effects of light adaptation on the mutant's excited state dynamics . . . 14
2.6 Computational Details . . . 16
2.6.1 Arch-3 QM/MM model, population dynamics and initial conditions . . . 16
2.6.2 Reaction quantum yield and excited state lifetime . . . 16
2.6.3 Time scaling of the Excited-State trajectory . . . 17

1 Methods

1.1 VIS-NIR femtosecond transient absorption spectroscopy

Transient absorption measurements were performed at room temperature with a home-built pump-probe set-up (60 fs time resolution) based on a 5kHz amplified Ti:Sapphire laser (Amplitude Technologies). The probe white-light continuum is generated in 2-mm Sapphire crystal that allows probing in the 460-1000 nm range. The 570 nm pump

[0a] *University of Strasbourg - CNRS, IPCMS, 23 Rue du Loess, 67034 Strasbourg, France*
[0b] *Department of Chemistry, Bowling Green State University, Bowling Green, OH 43403, USA*
[0c] *The Institute for Solid State Physics, University of Tokyo 5-1-5, Kashiwano-ha, Kashiwa, Chiba, 277-8581, Japan*
[0d] *Dipartimento di Biotecnologie, Chimica e Farmacia, Università di Siena, I-53100 Siena, Italy*
[0e] *LASIRE, Université de Lille, CNRS, 59000, Lille, France*
[0f] *LOB, CNRS, INSERM, École Polytechnique, Inst. Polytechnique de Paris, 91120 Palaiseau, France*

pulse was generated by an optical parametric amplifier (OPA) (TOPAS, Light Conversion) and polarized at the magic angle with respect to the probe beam polarization. The excitation energy density was set to $\sim 0.8\ mJ/cm^2$, which was verified to be in the linear range of excitation. The time delay between pump and probe was varied up to 1 ns by the optical delay stage. The 1.5 mL sample (OD 0.4-0.6/mm) was continuously illuminated with 505 nm LED and circulated through a 0.5 mm quartz flow cell using a peristaltic pump that allowed sample refreshment after each laser shot. The spectra were averaged over 6 scans for WT AR3 and 5 scans for DETC and ARCH-5.

1.2 Fluorescence up-conversion spectroscopy

Fluorescence up-conversion experiments were performed with a home-built set-up based on the same 5kHz amplified Ti:Sapphire laser[1]. It is optimised for $\sim$ 150 fs time resolution and broadband spectral detection. The 50-60 fs pump (570 nm) and the gate (1140 nm) pulses are produced at 5kHz repetition rate by an OPA (TOPAS, Light Conversion). The pump beam is focused on the sample placed in the 0.5 mm quartz flow cell (OD 0.4-0.6/mm), and the emitted fluorescence is collimated and focused into a non-linear crystal (BBO type II 0.4 mm) by the use of two parabolic off-axis mirrors. The gating pulse is delayed with respect to the pump by the means of a motorized delay line and focused into the non-linear crystal. Since we use type II sum frequency generation (SFG), the gate beam is polarized horizontally and the pump beam under magic angle with respect to the vertical. The time delay between pump and probe was varied up to 200 ps with 6 fs accuracy. With a fixed phase-matching angle the resulting SFG signal is guided towards a spectrometer and detected with a low-noise liquid-nitrogen cooled CCD camera. The 1.5 mL sample (OD 0.4-0.6/mm) was continuously illuminated with 505 nm LED and circulated through a 0.5 mm quartz flow cell using a peristaltic pump that allowed sample refreshment after each laser shot. The spectra were averaged over 3 or 5 scans of 3 or 5 s exposure time, respectively, for every delay point. Data post-processing (averaging, back conversion of fluorescence wavelength, background subtraction) was done using MATLAB software.

1.3 Raman spectroscopy

The Raman spectra were recorded with an FT- Raman spectrometer RFS 100/s (Bruker Optics) at room temperature. The excitation 1064 nm Nd:YAG laser power was set at 400mW. 1 ml of the sample (OD 0.4-0.6/mm) was placed in the 5 mm path length cell with a mirror back surface. Back-scattering geometry was used in the detection. The detection range was 50-3650 cm^{-1} with 4 cm^{-1} spectral resolution. The spectra were accumulated over 5000 scans to ensure a sufficient signal-to-noise ratio. The measurements of the samples in the light-adapted state were performed under continuous illumination by a 505 nm LED (37 nm FWHM).

1.4 Fluorescence spectroscopy

Steady-state fluorescence measurements were performed with an Edinburgh Instrument fluorescence spectrometer with an NIR-PMT 1200 nm detector at room temperature. Excitation and detection range wavelengths were varied for different samples. To determine the mutants' fluorescence quantum yields (Φ_f), the method introduced by Parker and Rees was used. The laser dyes Rhodamine 800 ($\Phi_{Fluo} = 0.250 \pm 0.027, \lambda_{exc} = 600nm$), Oxazine 170 = Oxazine 720 ($\Phi_{Fluo} = 0.579 \pm 0.032, \lambda_{exc} = 570nm$) and HITCI ($\Phi_{Fluo} = 0.283 \pm 0.017, \lambda_{exc} = 615nm$) dissolved in ethanol (OD<0.1/mm) were used as fluorescence standards [2]. The detection range for protein fluorescence was 650-1150 nm with a step of 1 nm and an acquisition time of 0.1 s. Excitation and detection slits were set to 3 nm and 4 nm, respectively. From 10 to 20 scans were averaged to have a sufficient signal-to-noise ratio.

1.5 UV-VIS Absorption spectroscopy

The steady-state UV-VIS absorption measurements were performed using LAMBDA 950 UV-VIS spectrometer (PerkinElmer) at room temperature. Spectral resolution 1 nm. 0.3 ml of the sample was placed in a 1 mm pathlength quartz cell.

1.6 Protein expression in *Escherichia coli* cells

Genes encoding WT AR3 and ARCH-5 were artificially synthesized with codon optimization for the *E. coli* expression (Genscript, NJ, USA) and cloned into NdeI–XhoI site of pET21a(+) vector (Novagen, Merck KGaA, Germany). The plasmid harboring the DETC mutant gene was constracted by site-directed mutagenesis using Quikchange® site-directed mutagenesis method (Agilent, CA, USA). The sequences of sense and antisense primers

used to make the mutant are GCGTTATGCGGAGTGGCTGTTCTGCACCCCGCTGCT and AGCAGCGGGGT-GCAGAACAGCCACTCCGCATAACGC, respectively. The constructed plasmids were transformed into *E. coli* C43(DE3) strain cells (Lucigen, WI, USA). The protein expression was induced by 0.1 mM isopropyl-β-D-thiogalactopyranoside (IPTG) in the presence of 10 μM ATR (Toronto Research Chemicals, Canada) for 4 h at 37 °C. A 6×His-tag was attached on the C-terminus of the expressed proteins.

1.7 Protein purification

Proteins were purified using a 5 mL Ni^{2+}-NTA column (HisTrap HP; Cytiva, Tokyo, Japan) on an ÄKTA start^{TM} protein purification system (Cytiva, MA, USA). The rhodopsin-expressing cells were harvested and resuspended in a buffer containing 50 mM Tris–HCl (pH 8.0) and 5 mM $MgCl_2$. The harvested cells were disrupted by sonication (Ultrasonic Homogenizer VP-300N, TAITEC, Japan). The membrane fraction was collected by ultracentrifugation (CP80NX, Eppendorf Himac Technologies, Japan) at 142,000 $\times g$ for 1 h. The proteins were solubilized in a buffer containing 50 mM Tris–HCl (pH 7.5), 300 mM NaCl, and 3% n-dodecyl-β-D-maltopyranoside (DDM). Solubilized proteins were separated from insoluble fractions by ultracentrifugation at 142,000 $\times g$ for 1 h. After loading the solubilized proteins on the Ni^{2+}-NTA column, the column was washed with a buffer containing 50 mM Tris–HCl (pH 7.5), 300 mM NaCl, 15 mM imidazole, and 0.1% DDM. The 6×His-tagged proteins were eluted using a 15 to 500 mM imidazole gradient with a buffer containing 50 mM Tris–HCl (pH 7.5), 300 mM NaCl, and 0.1% DDM. The eluted proteins were dialyzed using a buffer containing 20 mM HEPES–NaOH (pH 7.0), 100 mM NaCl, 0.05% DDM to remove imidazole. Absorption spectra were recorded with a UV–vis spectrometer (V-750, JASCO, Japan).

1.8 Retinal configuration analysis by HPLC

The retinal isomers of WT AR3 and its variants were analyzed using an HPLC system consisting of a silica column (3 μm particle size, 150 × 6.0 mm; Pack SIL, YMC, Japan), a pump (PU-4580, JASCO, Japan), and UV–vis detector (UV-4570, JASCO, Japan). Purified WT AR3 and its variants were solubilized in a buffer containing 20 mM HEPES (pH 7.5), 100 mM NaCl, 0.05% DDM. Before the measurements, samples underwent dark adaptation for 2 days at 4 °C. For analysis, a 75-μL sample was mixed with 280 μL of ice-cold 90% methanol and 25 μL of 2 M hydroxylamine (NH_2OH) to convert retinal chromophore into retinal oxime. The retinal oxime was extracted with 700 μL of *n*-hexane, and 200 μL of the solution was injected into the HPLC system. The solvent, containing 15% ethyl acetate and 0.15% ethanol in *n*-hexane, flowed at 1.0 $mL\,min^{-1}$. For measurements of the samples after illumination, the sample was exposed to light for 20 minutes using a Xe lamp with a bandpass filter (510 ± 5 nm). The extraction of retinal oxime was performed 30 seconds after turning off the light. The molar composition of the sample was calculated from the peak areas and the molar extinction coefficients at 360 nm (all-*trans*-15-*syn*: 54,900 $M^{-1}\,cm^{-1}$; all-*trans*-15-*anti*: 51,600 $M^{-1}\,cm^{-1}$; 13-*cis*-15-*syn*, 49,000 $M^{-1}\,cm^{-1}$; 13-*cis*-15-*anti*, 52,100 $M^{-1}\,cm^{-1}$; 11-*cis*-15-*syn*, 35,000 $M^{-1}\,cm^{-1}$; 11-*cis*-15-*anti*, 29,600 $M^{-1}\,cm^{-1}$; 9-*cis*-15-*syn*, 39,300 $M^{-1}\,cm^{-1}$; 9-*cis*-15-*anti*, 30,600 $M^{-1}\,cm^{-1}$).[3]

1.9 UV-VIS nanosecond transient absorption spectroscopy

The detail of the laser flash photolysis system was previously reported.[4,5] Purified WT AR-3, DETC, and ARCH-5 were solubilized in 20 mM MES–NaOH (pH 6), 0.05% DDM, 100 mM NaCl. The absorption of the protein solution was adjusted to ∼0.5 (total protein concentration ∼0.25 $mg\,mL^{-1}$) at the absorption maximum wavelength (559 nm). For the measurement of Φ_{AR-3}, we used light-adapted bR as a reference sample and its absorption was adjusted to match with that of the LA WT AR-3 at the excitation wavelength (532 nm) with an error less than 1%. The LA WT AR-3 was exposed to the second harmonics beam from a nanosecond-pulsed Nd:YAG laser ($\lambda = 532nm$, 0.25–8.2 $mJ\,cm^{-2}$, 3.33 Hz) (INDI40, Spectra-Physics, CA, USA), while DETC and ARCH-5 were excited at 600 nm (5.3 $mJ\,cm^{-2}$, 1 and 3.33 Hz, respecitvely) (basiScan, Spectra-Physics, CA, USA). The transient absorption spectra at specific time points were obtained by monitoring the intensity change of a Xe arc lamp (L9289-01, Hamamatsu Photonics, Japan) with an ICCD linear array detector (C8808-01, Hamamatsu Photonics, Japan). For each experiment, 150 spectra were recorded and averaged. The time evolution of the transient absorption change at specific probe wavelength was obtained by observing the intensity change of the output of a Xe arc lamp (L9289-01, Hamamatsu Photonics, Japan), monochromated by a monochrometer (S-10, SOMA OPTICS, Japan) and passed through the sample, after photo-excitation by a photomultiplier tube (R10699, Hamamatsu Photonics, Japan) equipped with a notch filter (532 nm, bandwidth = 17 nm) (Semrock, NY, USA) to remove the scattered laser beam. To increase the signal-to-noise (S/N) ratio, 200–400 signals were averaged. The signals of the WT AR3 were global-fitted with a multi-exponential function to determine the lifetimes of each photointermediate.

1.10 QM/MM model construction and validation

As previously reported[6,7], a hybrid quantum mechanics molecular mechanics (QM/MM) model of AR-3 in its S_0 state and suitable for reactivity studies can be constructed by employing the automated *a*-ARM protocol[6,8] that uses the available protein crystal structure (PDB ID: 6GUX)[9] as the input. The resulting model is a gas-phase monomeric model neutralized with external counterions that predicts a ≈30 nm blue shifted λ^a_{max} with respect to the experimental data, in line with the methodology error-bar[8]. Here, we further validate the model by computing the absorption band of AR-3. This is achieved by simulating the S_0 room temperature Boltzmann distribution of the protein with 200 snapshots generated using a protocol already detailed in the literature (see for instance ref.[10] and Supporting Information). As shown in Figure S1, the obtained λ^a_{max} of 560 nm is only 6 nm red-shifted with respect to the experimental observation.

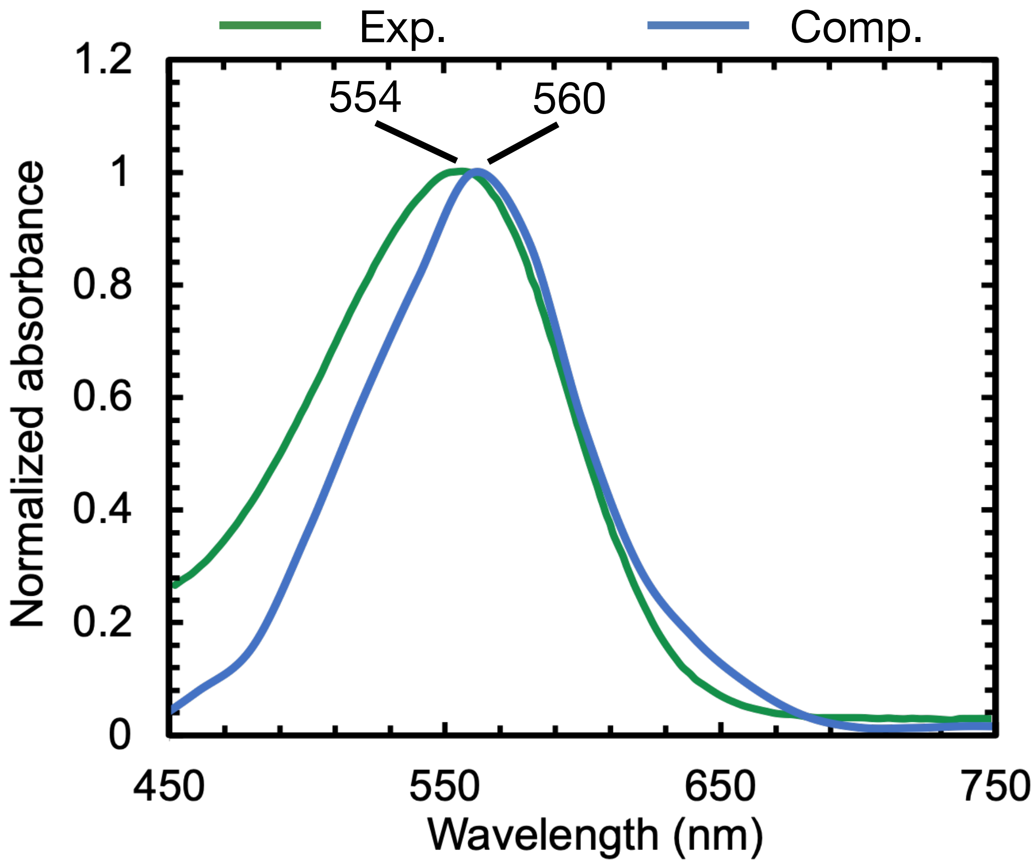

Figure S1: Computed (blue) and experimental (green) absorption band of AR-3 WT.

Such agreement indicates that the constructed model correctly describes the interaction between the complex electrostatic interaction environment generated by the opsin cavity and the electronic structure of the all-*trans* chromophore. All potential energy surfaces (PESs) were computed using such QM/MM model featuring a complete active space self-consistent field (CASSCF) wavefunction and the 6-31G* basis set (CASSCF/6-31G*) to treat the QM part or photoactive chromophore (Figure 8B main text). To evaluate the potential energies necessary for comparison with the experimental data, single point rotated multi-state complete active space second order perturbation theory (RMS-CASPT2) calculations[11,12], were then performed on the CASSCF structures using the same basis set. In specific cases, as described above, also an RMS-CASPT2 geometry optimization was carried out to determine the AR-3 equilibrium structure.

1.11 Non-adiabatic trajectory computation

A swarm of 200 quantum-classical trajectories, driven by gradients computed at the CASSCF/6-31G* level and based on the QM/MM model above, were propagated starting on the S_1 PES using the Tully's fewest switches surface hop algorithm to account for the non-radiative deactivation of AR-3 and the successive S_0 photoproduct formation

(see Supporting Information for details). We found that the all-*trans* chromophore of AR-3 isomerizes exclusively at the C13=C14 double bond (see Figure 8C). The simulation did not detect other alternative isomerization processes. This method also identifies the reaction coordinate, namely coupled hydrogen and methyl out-of-plane motion at C14 and C13 leading to the CoIn.

2 Supplementary data

2.1 Fluorescence up-conversion spectroscopy, FLUPS

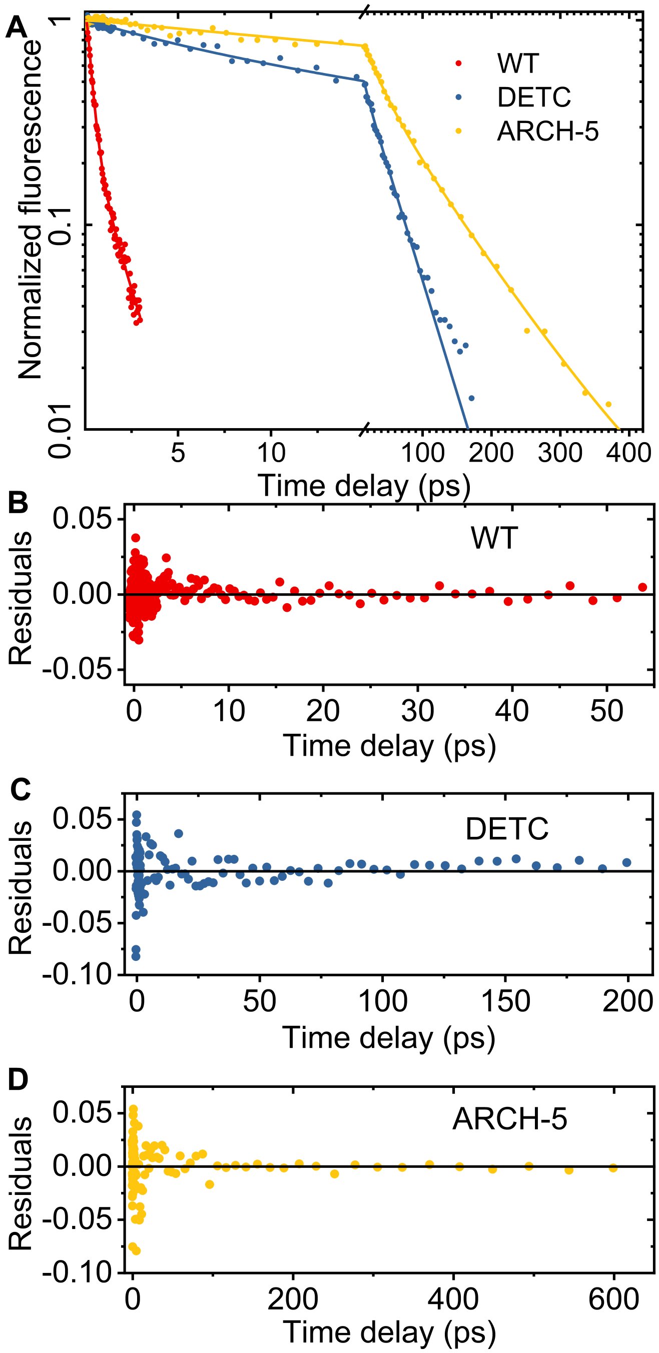

Figure S2: FLUPS results. (A) Comparison of the normalized fluorescence decays between WT, DETC and ARCH-5 (dots) in semi-logarithmic coordinates. Kinetics were averaged over 15 nm around the fluorescence maximum. As we can see fluorescence kinetics of each of the samples have two linear slopes. Solid lines present the best result obtained for fit with the bi-exponential (3-exponential for ARCH-5) decay function. Panels (B), (C) and (D) present the difference between data and fit as function of delay time for WT, DETC and ARCH-5 respectively.

2.2 Average fluorescence lifetimes and quantum yield

The fluorescence QY is defined as

$$\Phi_f = FQY = \frac{nb \text{ of emitted ph}/s}{nb \text{ of absorbed ph}/s} \tag{S1}$$

Experimentally, it is determined by measuring the temporally and spectrally integrated fluorescence signal $\int_{\lambda_{em}} F(\lambda)d\lambda$ with respect to the one of a reference sample, with a known FQY, and normalising with respect to the different amount of absorption at the excitation wavelength $f(\lambda_{ex})$:

$$\Phi_f^u = \Phi_f^s \frac{f_s(\lambda_{ex})}{f_u(\lambda_{ex})} \frac{\int_{\lambda_{em}} F^u(\lambda)d\lambda}{\int_{\lambda_{em}} F^s(\lambda)d\lambda} \frac{n_u^2}{n_s^2}, \tag{S2}$$

where u and s denote the properties of the "unknown" sample and the reference sample, respectively. Since the solvents are the same, the ratio of refractive indices n_u and n_s is 1. Exactly this approach was used for determining the Φ_f's of the mutants with HITCI as reference sample. The absorption at the excitation wavelength λ_{ex} is related to the absorbance A by :

$$f_{(\cdot)}(\lambda_{ex}) = 1 - 10^{-A_{(\cdot)}(\lambda_{ex})} \tag{S3}$$

When we compare the Φ_f of the two mutants, it boils down to comparing the temporally and spectrally integrated fluorescence signals F, weighted by the absorption factor:

$$\frac{\Phi_f^{ARCH-5}}{\Phi_f^{DETC}} = \frac{f_{DETC}(\lambda_{EX})}{f_{ARCH-5}(\lambda_{EX})} \frac{\int_{em} F^{ARCH-5}(\lambda)d\lambda}{\int_{em} F^{DETC}(\lambda)d\lambda} \tag{S4}$$

where the integral is carried out over the entire wavelength range of emission. How does the temporally integrated fluorescence signal $\int_{em} F^u(\lambda)d\lambda$ relate to the time-resolved kinetics ? It is proportional to the time integral over the kinetics:

$$\int_{em} F^u(\lambda)d\lambda = C \int_0^\infty I_0 \left(\sum_{i=1}^N A_i e^{-t/\tau_i} \right) dt \tag{S5}$$

for a multi-exponential decay. C is a proportionality factor describing the sensitivity of the instrument, and I_0 is the signal at t=0, which is proportional to the exc. power, the absorption $f(\lambda_{ex})$, and the radiative rate of the fluorophore. The temporal integral goes over the sum of exponentials and is simply given by $\int_0^\infty \left(\sum_{i=1}^N A_i e^{-t/\tau_i} \right) dt = \sum_{i=1}^N A_i \tau_i$, since $\int_0^\infty A_i e^{-t/\tau_i} dt = A_i \tau_i$. When we now relate the ratio of the time-integrated fluorescence signals to the temporal integral of kinetics we find

$$\frac{\int_{em} F^{ARCH-5}(\lambda)d\lambda}{\int_{em} F^{DETC}(\lambda)d\lambda} = \frac{\left[\int_0^\infty I_0 \left(\sum_{i=1}^N A_i e^{-t/\tau_i} \right) dt\right]_{ARCH-5}}{\left[\int_0^\infty I_0 \left(\sum_{i=1}^N A_i e^{-t/\tau_i} \right) dt\right]_{DETC}} = \frac{I_0^{ARCH-5}}{I_0^{DETC}} \frac{[\sum_{i=1}^N A_i \tau_i]_{ARCH-5}}{[\sum_{i=1}^N A_i \tau_i]_{DETC}} \tag{S6}$$

The average lifetimes calculated in the paper are $\langle \tau_{ARCH-5} \rangle = [\sum_{i=1}^N A_i \tau_i]_{ARCH-5}$ and $\langle \tau_{DETC} \rangle = [\sum_{i=1}^N A_i \tau_i]_{DETC}$ since $\sum_{i=1}^N A_i = 1$. Hence, we have

$$\frac{\int_{em} F^{ARCH-5}(\lambda)d\lambda}{\int_{em} F^{DETC}(\lambda)d\lambda} = \frac{I_0^{ARCH-5}}{I_0^{DETC}} \frac{\langle \tau_{ARCH-5} \rangle}{\langle \tau_{DETC} \rangle} \tag{S7}$$

Now, back to the ratio of the Φ_f 's:

$$\frac{\Phi_f^{ARCH-5}}{\Phi_f^{DETC}} = \frac{f_{DETC}(\lambda_{EX})}{f_{ARCH-5}(\lambda_{EX})} \frac{\int_{em} F^{ARCH-5}(\lambda)d\lambda}{\int_{em} F^{DETC}(\lambda)d\lambda} = \frac{f_{DETC}(\lambda_{EX})}{f_{ARCH-5}(\lambda_{EX})} \frac{I_0^{ARCH-5}}{I_0^{DETC}} \frac{\langle \tau_{ARCH-5} \rangle}{\langle \tau_{DETC} \rangle} \tag{S8}$$

Since I_0 scales a) with the absorption $f(\lambda_{ex})$, and b) the exc. power was the same for both samples, and it follows

$$\frac{\Phi_f^{ARCH-5}}{\Phi_f^{DETC}} = \frac{\langle \tau_{ARCH-5} \rangle}{\langle \tau_{DETC} \rangle} \tag{S9}$$

under the assumption that DETC and Arch-5 have the same radiative rates. The latter assumption might contribute to the fact that the ratios reported in Table 1 (main text) are not exactly the same. Note that the above considerations apply for any multi-exponential decay, and in particular for the mixture of isomers present in the mutants.

2.3 Transient Absorption Spectroscopy

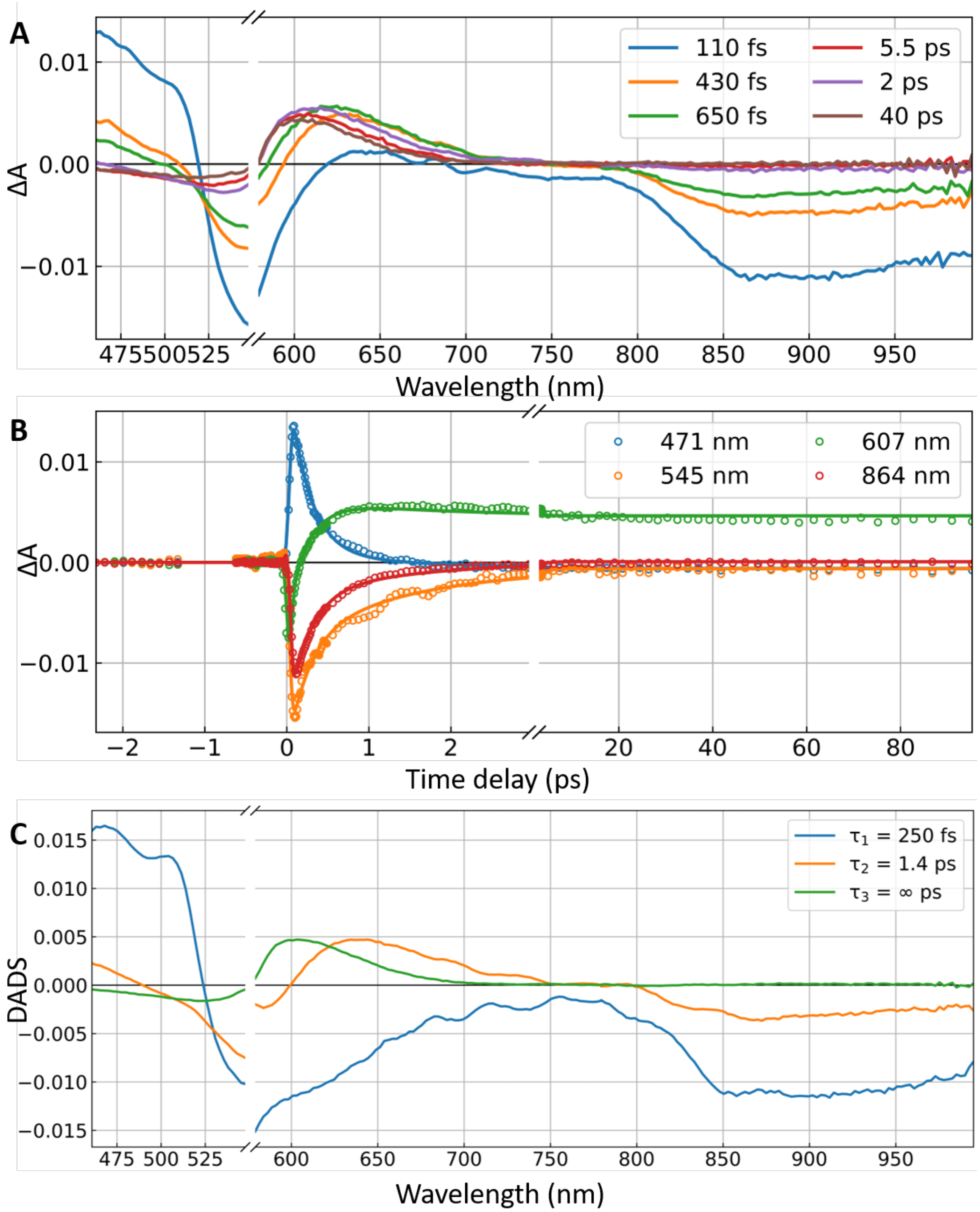

Figure S3: TAS results for WT AR-3. (A) Differential absorption spectra of WT AR-3 at the time delays indicated in the legend after the 570 nm photoexcitation. (B) dots: kinetics at selected wavelengths, solid lines: kinetics obtained as the result of the global fit. (C) decay-associated difference spectra obtained as the result of the global fit

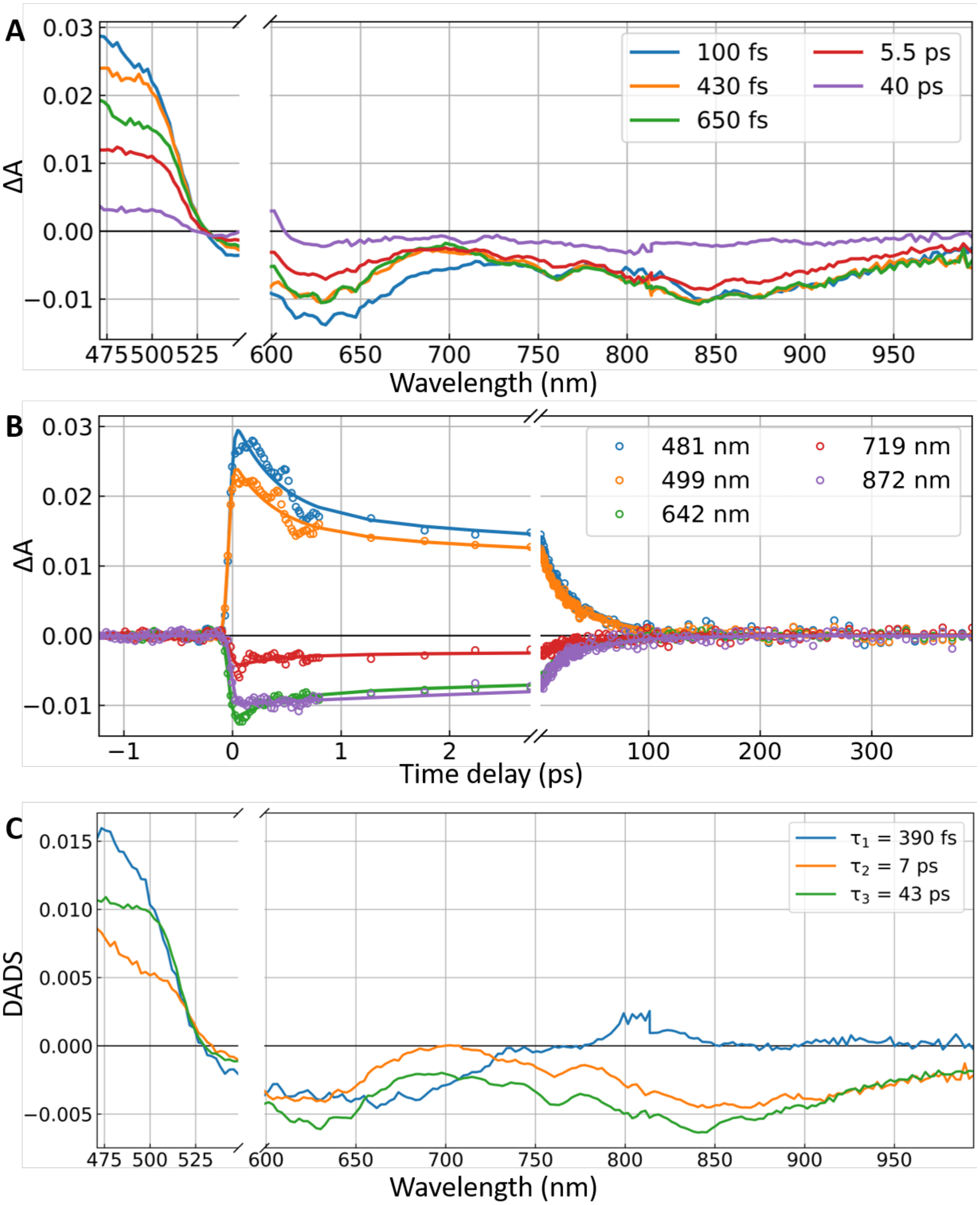

Figure S4: TAS results for DETC. (A) Differential absorption spectra of DETC at the time delays indicated in the legend after the 570 nm photoexcitation. (B) Dots: Kinetics at selected wavelengths (cf. legend), solid lines: kinetics obtained as the result of the global fit. (C) Decay-associated difference spectra obtained as the result of the global fit

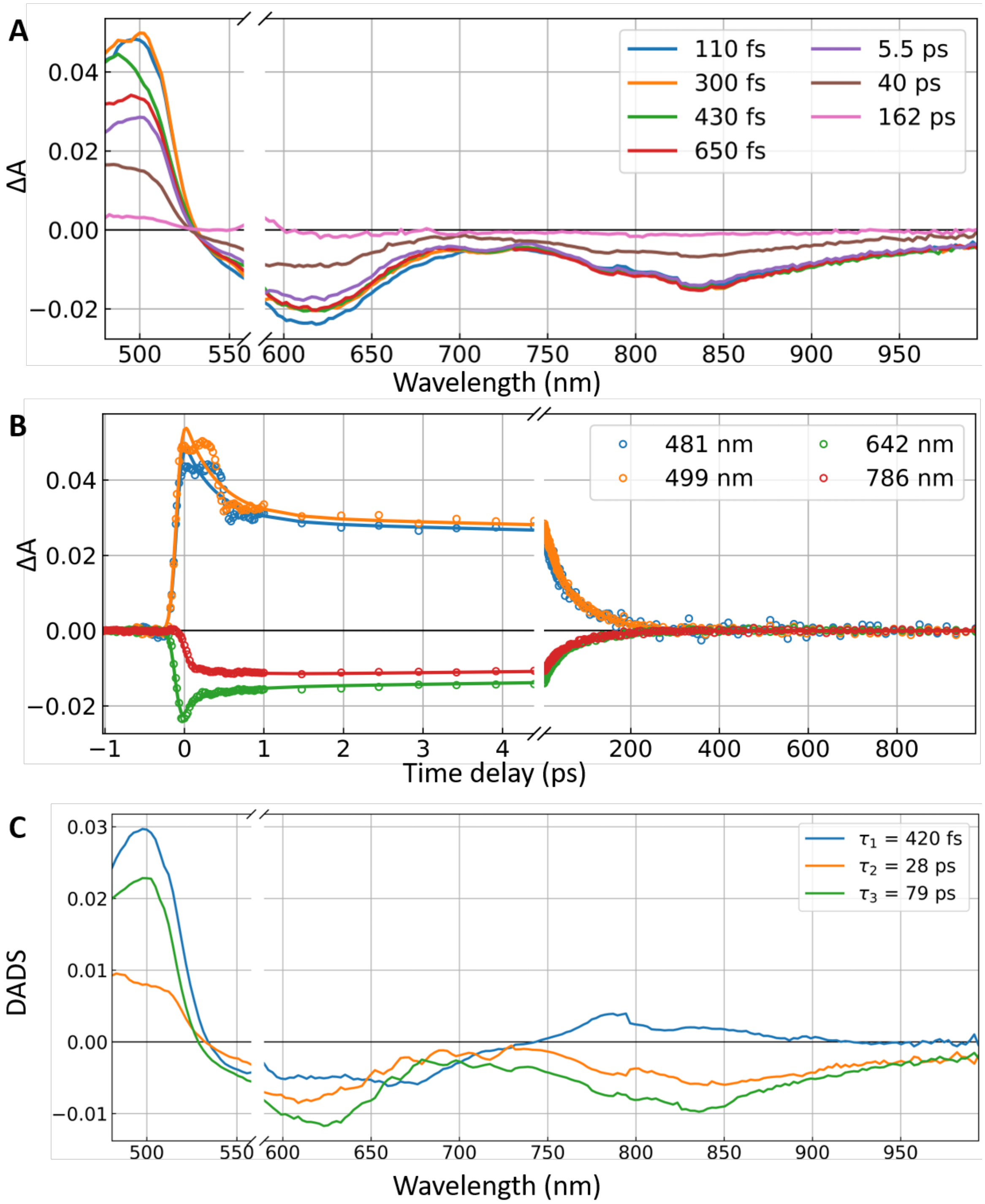

Figure S5: TAS results for ARCH-5. (A) Differential absorption spectra of ARCH-5 at the time delay pointed in the legend after 570 nm photoexcitation. (B) Dots: Kinetics at selected wavelengths (cf. legend), solid lines: kinetics obtained as the result of the global fit. (C) Decay-associated difference spectra obtained as the result of the global fit

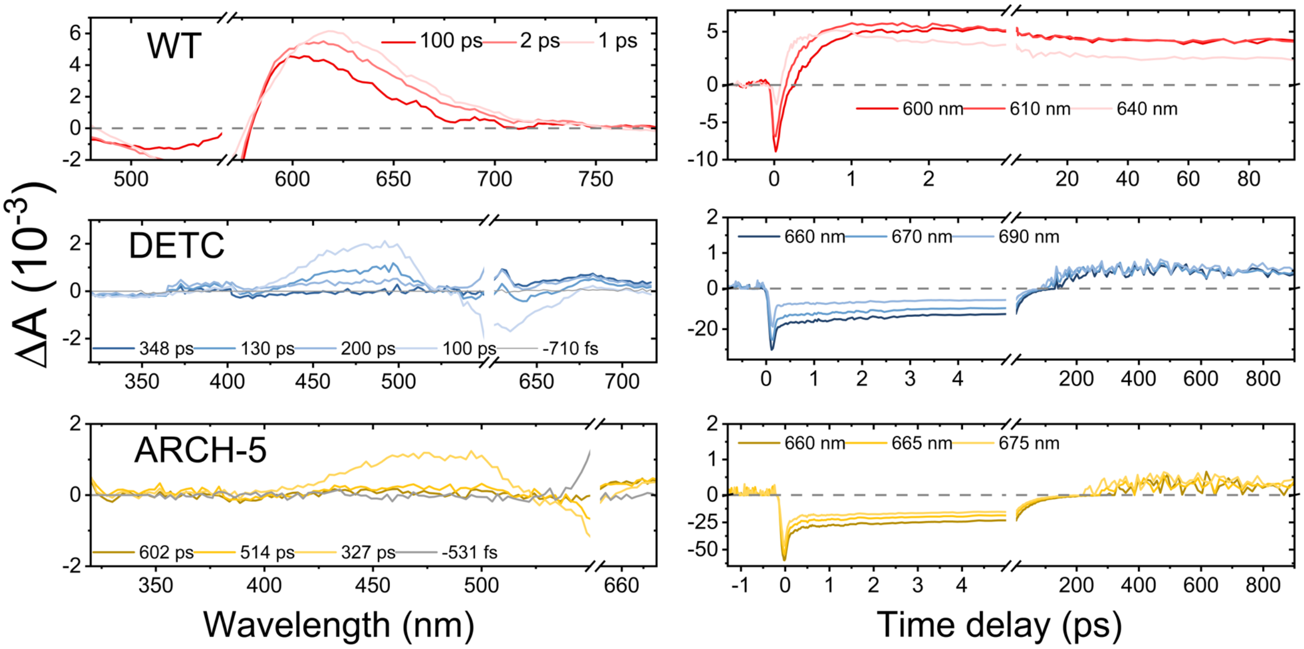

Figure S6: Results of TAS experiments at higher excitation energy density $\sim 1.3\ mJ/cm^2$. Left: Transient absorption signal at long time delays (cf. legend) after 560 nm excitation for WT (top red) and after 610 nm excitation for DETC (middle blue) and ARCH-5 (bottom yellow) at pH 6. For WT strong negative and positive signals are observed at 520 and 610 nm respectively. The positive signal is due to the K photoproduct and the negative signal is the depopulation of the ground state (GSB) due to photoproduct formation. For DETC and ARCH-5 positive signals in the 400-550 nm range correspond to the excited state absorption, and the negative signal around 650 nm is GSB. Low K photoproduct signals are observed for mutants in the region above 650 nm. Right: Kinetics of the formation of K photoproduct of WT (top), DETC (middle) and ARCH-5 (bottom) at the wavelengths indicated in the legend. The strong negative signal at the start of the kinetics caused by GSB decays on a 0.2 ps range for WT and a 100-200 ps range for mutants followed by the positive transient absorption signal of the photoproduct.

2.4 UV-VIS nanosecond transient absorption spectroscopy

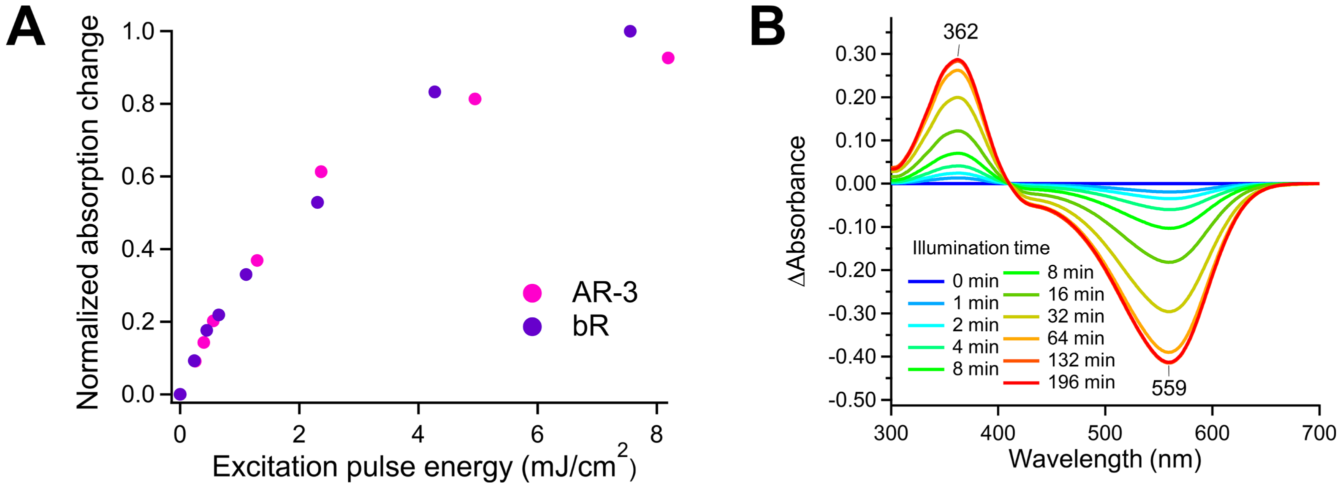

Figure S7: (A) Excitation pulse energy dependence of transient absorption signal of the wt AR-3 and bR. (B) Differential absorbance change upon the hydrolysis reaction of the RSB releasing retinal oxime showing the 362-nm absorption peak.

2.5 Light adaptation effect

2.5.1 Protocol and effects of light adaptation on the steady-state absorption spectra

The effect of light adaptation on the absorption spectra was studied by steady-state UV-VIS spectroscopy. The samples were exposed to a 505 nm LED with an intensity of 15 $mW \cdot cm^{-2}$ at the sample position. The effect of light adaptation on WT is presented in Figure S8. For WT after 20 min of illumination, an absorption decrease in the 300–500 nm range and a red-shift and rise of the main band peaking at 559 nm were observed. Longer 1H exposure does not have a significant influence on the spectrum. After the excitation light switch-off, the thermal recovery to the dark state is observed. The spectral red shift under the 505 nm illumination is assigned to the light conversion of 13-*cis*-15-*syn* isomers to the all-*trans*-15-*anti* form in agreement with the HPLC analysis and FT-Raman experiments in the dark and light-adapted states. The same effect of light adaptation was observed for DETC. However, back recovery in the dark is much faster for DETC than for WT. A decrease in the absorption in the main peak and a rise of the absorption at 390 nm is assigned to a partial formation of the retinal isomer with a deprotonated retinal Schiff base (RSB) [13]. In contrast to the WT and DETC samples, no spectral shift is observed as a result of light adaptation for ARCH-5. Light adaptation causes a slight increase of RSB (absorption decreases at the main peak and rises at 390 nm).

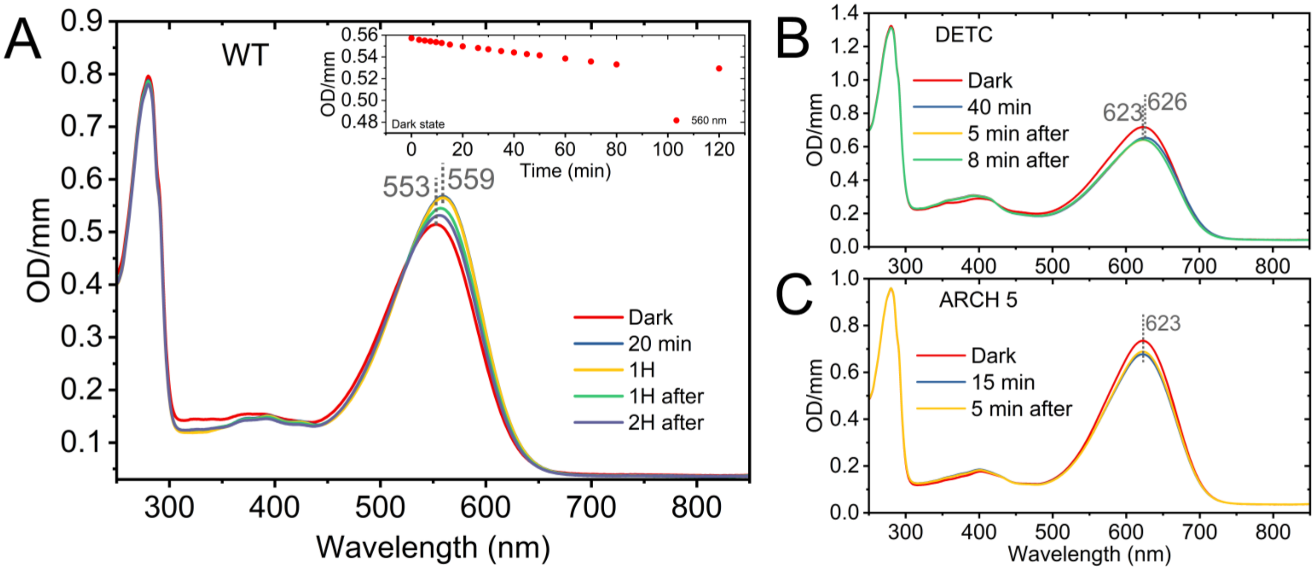

Figure S8: Effects of light adaptation on the steady-state absorption spectra at pH6. The evolution of the WT (A), DETC (B) and ARCH 5 (C) absorption spectra under illumination. The red line shows the absorption spectra before light exposure (dark-adapted state). (A) The absorption spectra recorded 1H (green line) and 2H (purple line) after the excitation light switch-off are presented. The inset shows the evolution of absorbance of WT at 560 nm after the excitation light is switched off over 2H (incomplete recovery, in the dark at room temperature).

2.5.2 Effects of light adaptation on the mutant's excited state dynamics

To study the effect of isomer composition on the excited-state dynamics of mutants TAS measurements were performed on the dark and light-adapted form of DETC and ARCH at pH 6. Figure S9 shows obtained kinetics in the ESA region. Only minor changes in the kinetics are observed as an effect of light adaptation. To analyse tehFor DETC as a result of the fit three time constants $\tau_1 = 8$ ps, $\tau_2 = 34$ ps and $\tau_3 = 105$ ps were obtained. The first two time constants are assigned to the excited state decay of all-trans and 13-cis retinal respectively and the 100 ps one is caused by GSB spectral relaxation. To account for the change of the isomer composition due to light adaptation we change the relative amplitudes in the 3 exponential decay fit of the dark-adapted sample: the amplitude of 8 ps time constants is scaled proportionally to the change of the all-trans retinal concentration as result of light adaptation (from 22% to 29%) and relative amplitude of 34 ps is scaled proportionally to the change of 13-cis retinal concentration (from 77% to 65%). The result of the scaling is presented in the semitransparent violet line. We see that the obtained line describes closely the kinetics of the light-adapted sample. The opposite scaling was done to fit the light-adapted sample (semi-transparent blue line).

In the case of ARCH-5, light and dark-adapted kinetics were fitted with 2-exponential decay with time constants of $\tau_1 = 30$ ps and $\tau_2 = 91$ ps. These time constants are assigned to the excited state decay of all-trans and 13-cis retinal respectively. Similarly to DETC, scaling for the change of the isomer composition in ARCH-5 was made. The semi-transparent violet line is obtained by the following change on the relative amplitudes in the 2 exponential decay fit of the dark-adapted sample: the amplitude of 30 ps time constants is scaled proportionally to the change of the all-trans retinal concentration as the result of light adaptation (from 22% to 30%) and relative amplitude of 91 ps is scaled proportionally to the change of 13-cis retinal concentration (from 76% to 64%). The result of scaling has a trend to describe the light-adapted kinetics but the fit is not as good. It could be caused by the fact that the dark-adapted sample is already partially light-adapted by the pump pulses. The opposite scaling was done to the fit of the light-adapted sample to obtain a semi-transparent blue line, which tends to describe the dark-adapted sample well.

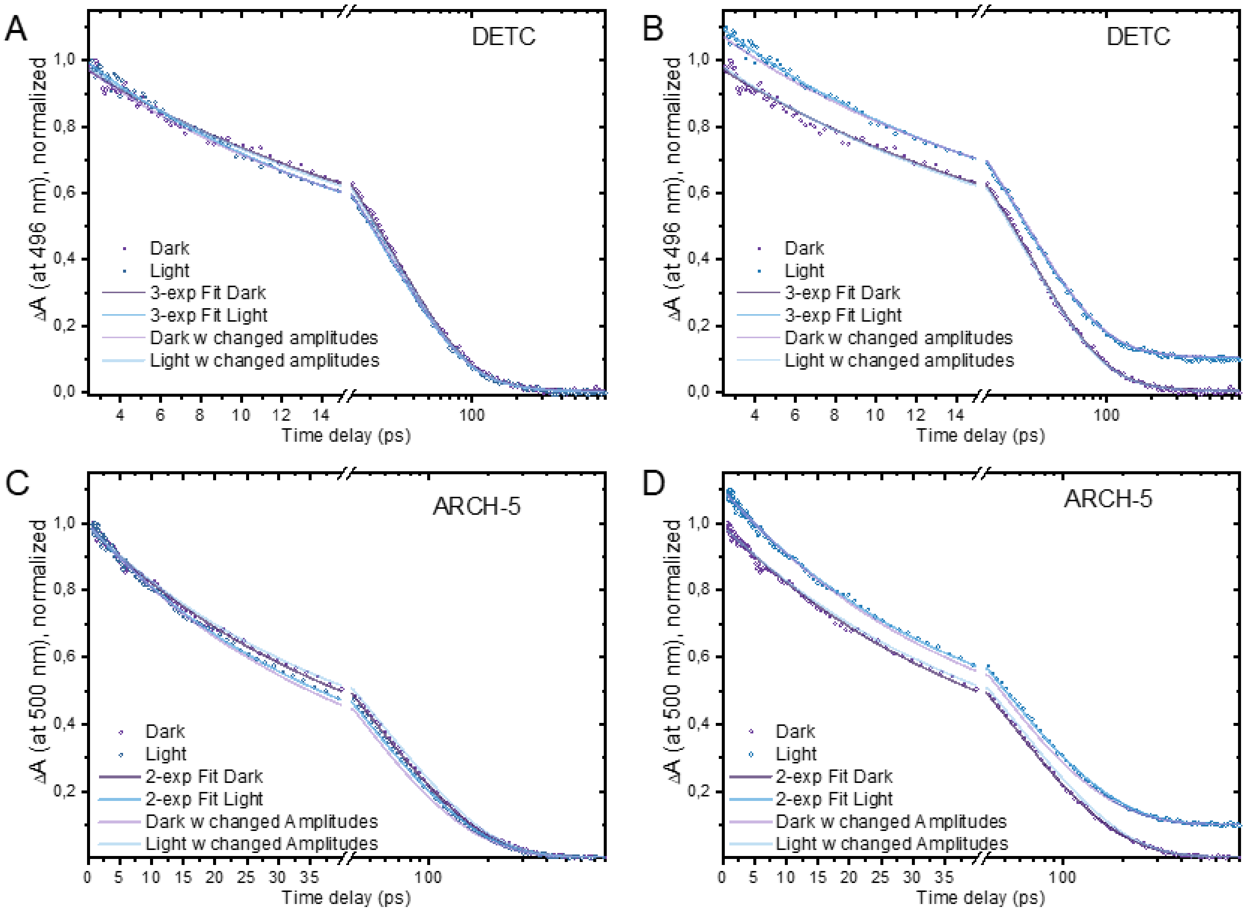

Figure S9: Light adaptation effect on TA kinetics of DETC (A-B) and ARCH-5 (C-D) mutants. (A) Open dots represent the normalized experimental data of dark-adapted (violet) and light-adapted (blue) samples at 496 nm. The bright violet and blue lines correspond to the 3-exponential decay fit of the experimental data in the dark and light-adapted forms, respectively. The semi-transparent violet line is obtained by changing only the relative amplitudes in the 3-exponential fit of the dark-adapted sample. (C) The scattered dots represent the normalized experimental data of dark-adapted (violet) and light-adapted (blue) Arch-5 at 500 nm (ESA region). The bright violet and blue lines correspond to the 2-exponential decay fit of the experimental data in the dark and light-adapted forms, respectively. The semi-transparent violet and blue lines are obtained by changing only the relative amplitudes in the 2-exponential decay fit of the dark- and light-adapted sample. Panels B and D present the same data as A and C respectively with the light-adapted kinetics vertically shifted by 0.1 to avoid data overlap.

2.6 Computational Details

2.6.1 Arch-3 QM/MM model, population dynamics and initial conditions

Construction of a QM/MM model for WT AR-3 was carried out using the a-ARM protocol[6,8], starting from the 1.30 Å resolution crystallographic structure found in the Protein Data Bank (PDB ID 6GUX)[9] as input, following a previously established protocol[7]. Within this model, the QM layer comprises the atoms of the retinylidene chromophore, NH group, and $C_\epsilon H_3$ substituent, treated at the CASSCF level of theory with a 6-31G* basis. The active space consists of 12 electrons in 12 orbitals, encompassing the entire π-system of the retinal chromophore moiety and defining a multiconfigurational wavefunction approach. The MM layer, composed of the remaining protein atoms including those of the $C_\delta H_2$ moiety, belonging to the Lys226 side-chain, is described by a modified AMBER94 force field[14] incorporating specific Lys296 side-chain parameters[15]. During trajectory calculations, side-chains and water molecules with at least one atom within 4 of any retinal chromophore atom were kept flexible, while all other atoms remained frozen. Initial conditions for ground state populations at room temperature were generated through a protocol described previously[16]. This included initiating a 20 ns MM dynamics simulation at 298K from an S_0 optimized geometry derived from a-ARM protocol which reproduces the corresponding vertical excitation energy relative to the maximum absorption wavelength λ^a_{max}. Subsequently, 200 snapshots (geometries and velocities) were extracted during the dynamics at 100 ps intervals following a suitable equilibration period (200 fs). From these snapshots, 200 HF/6-31G*/AMBER trajectories were propagated for 200 fs, followed by the corresponding S_0 2-roots state-average CASSCF/6-31G*/AMBER for 50 fs. The geometries and velocities of the final snapshot of the 50 fs propagation were assumed to represent the Boltzmann distribution, serving as initial conditions for subsequent trajectory computations on S_1. All calculations were performed using the Molcas[17]/Tinker package[18].

Property	λ^a_{max}, nm	lifetime, fs	IQY
Exp.	554	340	0.54
Comp.	560	191	0.63

Table S1: Comparison between the observed experimental parameters and the computational ones of WT AR-3.

We evaluated the λ^a_{max} value from the absorbance band (Fig. S1) computed at the 3-root-state-average CASPT2/CASSCF/6-31G*/Amber level of theory. As reported in Table S1, the computed 556 nm value closely agrees with the observed value of 560 nm.

2.6.2 Reaction quantum yield and excited state lifetime

The excited-state quantum-classical trajectories were propagated at the 2-root-state-average CASSCF/6-31G*/ Amber level of theory with a time step of 1 fs. The S_1 to S_0 hop event is determined by using the Tully surface-hop method[19] including the decoherence corretion[20] implemented in the Molcas[17]/Tinker package[18]. (A test supporting the validity of the Tully surface-hopping method for alkylated or protonated Schiff bases has been recently reported.[16]) The reaction quantum yield (IQY) was calculated as the ratio of the number of trajectories forming the photoproduct (i.e this is determined by looking the α value at the last step) over the total number of decayed trajectories. The predicted and observed values are 0.63 and 0.54, respectively. In order to determine the excited state lifetime (τ) reported in Table S1, we have used the following fitting formula comprising a lag time and two exponential decay times.

$$f(t) = a_1 e^{(-(\frac{t-t_1}{t_2})^2)} + y_0 + ((1-a_1) - y_0)e^{(\frac{-(t-t_1)}{t_3})} \qquad Eq.\ 1^{21}$$

$$\tau = t_1 + a_1 t_2 + (1 - a_1 - y_0)t_3 \qquad Eq.\ 2^{21}$$

Where a_1 represents the weight of the Gaussian decay component, y_0 is a fitting parameter (fraction of molecules that remain in the excited state relative to the initial population after 400 fs), t_1 refers to the latency time (the period before the onset of decay), and t_2 and t_3 correspond to the decay times for the Gaussian and exponential components, respectively. Fitting the S_1 population decay with this Gaussian-plus-exponential equation (Eq. 1), 0.71, 0, 67 fs, 122 fs and 128 fs values were acquired for a_1, y_0 t_1, t_2 and t_3, respectively. The computed τ from Eq. 2 is 191 fs which should be compared with the observed 340 fs value. In conclusion, the values of λ^a_{max}, IQY and lifetime computed based on AR-3 WT QM/MM model and population dynamics indicate a semi-quantitative agreement with the experimental observations (Table S1). These properties have been computed, limiting the simulation to 200 trajectories (due to the high computational cost) .

2.6.3 Time scaling of the Excited-State trajectory

There is a significant difference (∼150 fs) when considering the computed and observed τ values. We notice that this could, in part, depend on factors such as the limited accuracy of the potential energy surface (PES) and forces calculated at the CASSCF level that miss a significant portion of dynamic electron correlation energy. Previous studies indicate that including dynamic electron correlation results in a flatter excited state PES, whereas the CASSCF method tends to systematically produce steeper excited state PESs[22]. To confirm this discrepancy, the energies of the S_1 PES at the Franck-Condon (FC) point, S_1 minimum, and CoIn were obtained for the optimized FC geometry at both the CASSCF level and the RMS-CASPT2 level. It was previously suggested that this kind of FC trajectory is a useful probe to capture the average behavior of a wavepacket[23].

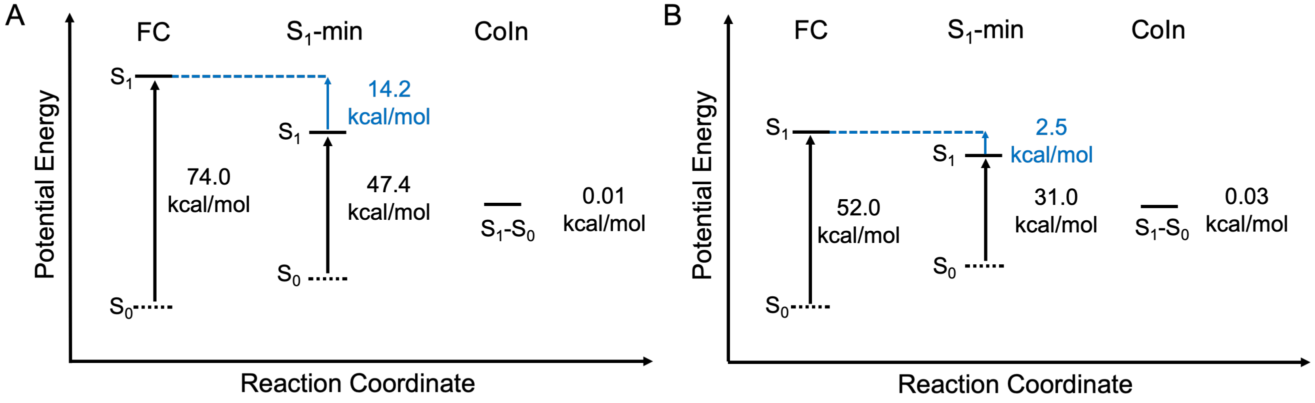

Figure S10: Potential energy diagrams of FC geometry of AR-3 WT at FC point, S_1 minima and CoIn for both A. at CASSCF level B. RMS-CASPT2 level.

In Figure S10, the potential energy gaps between the S_1 states at the FC point and the S_1 minima are greater at the CASSCF level compared to the RMS-CASPT2 level. This indicates that the excited state PES is steeper at the CASSCF level and flatter at the RMS-CASPT2 level agreeing with the above statement. Even though RMS-CASPT2 calculations can be used to solve this problem, propagating hundreds of RMS-CASPT2 driven quantum-classical trajectories is still computationally unpractical. Therefore, a correction factor was derived by performing single-point RMS-CASPT2 calculations for the geometries at each femto second extracted by propagating a FC structure with zero initial velocity at the CASSCF level. The following equation can express the relationship between the CASSCF PES and the RMS-CASPT2 PES[24].

$$\nabla E_{RMS-CASPT2}(\overline{x}) \approx \alpha \nabla E_{CASSCF}(\overline{x}) \qquad Eq.\ 3^{24}$$

This relationship indicates that the S_1 CASSCF gradient is proportional to the RMS-CASPT2 gradient (through the factor α) within the studied region of the PES. The value of the scaling factor α is obtained by linear fitting of the corresponding energies (in Figure S11 we have chosen the relative values referred to as the ground-state minima). Accordingly, we obtained $\alpha = 0.634$, intercept = 8.896 and r = 0.958 from this linear plot. The energy calculation along the reaction coordinate enables a global scaling of the S_1 CASSCF PES, which is anticipated to match the shape of the RMS-CASPT2 energy surface, at least within the region explored by the computed trajectory. Since the scaled CASSCF and RMS-CASPT2 trajectories must align, we can establish a formal relationship between the unscaled CASSCF and RMS-CASPT2 gradients by scaling the time according to the following equation.

$$t = \alpha^{\frac{1}{2}} t' \qquad Eq.\ 4^{24}$$

The unscaled CASSCF and RMS-CASPT2 trajectories become equal as a function of a parameter t and t', respectively (t is the unphysical CASSCF time and t' is the physical RMS-CASPT2 time and α is the scaling factor).

$$\mathrm{t^{RMS-CASPT2} = a_{scale} \cdot t^{CASSCF}} \qquad Eq.\ 5$$

According to the α value, the scaled τ value was 240 fs. Still, there is 100 fs discrepancy in the computed τ value when compared to the experimental τ value. A potential reason for this discrepancy could be that in single-point calculations, geometries with zero kinetic energy are considered, which results in the loss of significant dynamic information associated with other geometries present in the experiments carried out at room temperature.

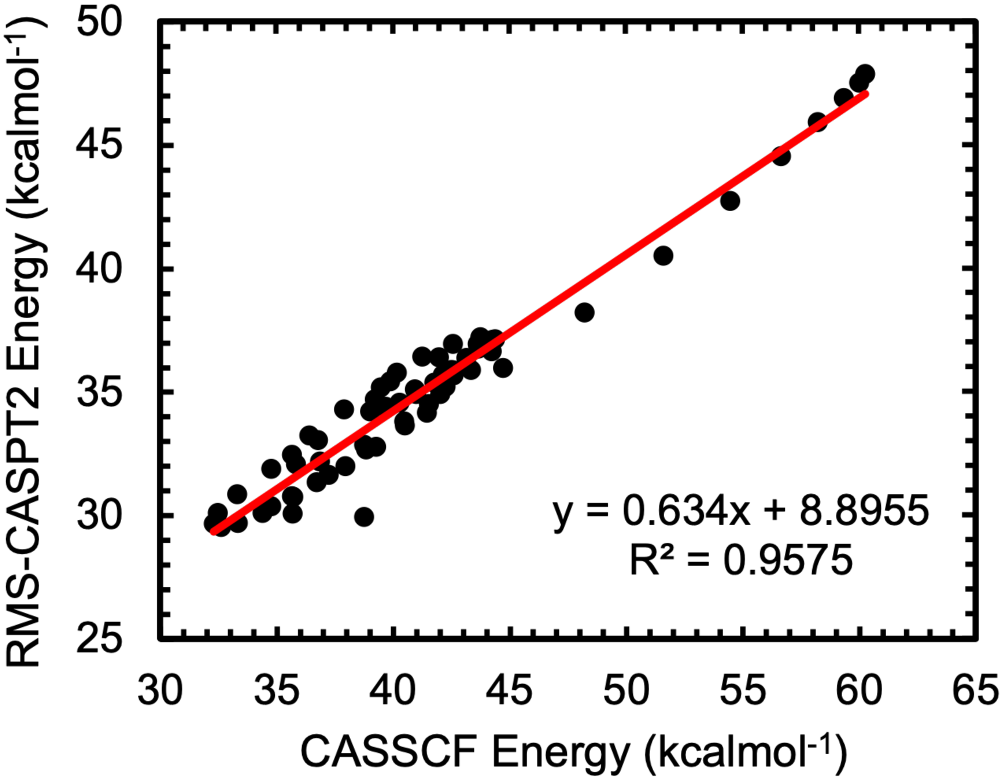

Figure S11: Linear fit of relative energies (refer to the ground state minima) determined at RMS-CASPT2 and CASSCF level of theory for the computed CASSCF trajectory.

References

[1] J. Léonard, T. Gelot, K. Torgasin and S. Haacke, *9th International Conference on Photonics and Imaging in Biology and Medicine (Pibm 2010)*, 2011, **277**, year.

[2] K. Rurack and M. Spieles, *Anal. Chem.*, 2011, **4**, 1232–1242.

[3] A. Trehan, R. S. H. Liu, Y. Shichida, Y. Imamoto, K. Nakamura and T. Yoshizawa, *Bioorg. Chem.*, 1990, **18**, 30–40.

[4] K. Inoue, H. Ono, R. Abe-Yoshizumi, S. Yoshizawa, H. Ito, K. Kogure and H. Kandori, *Nat. Commun.*, 2013, **4**, 1678.

[5] K. Inoue, S. P. Tsunoda, M. Singh, S. Tomida, S. Hososhima, M. Konno, R. Nakamura, H. Watanabe, P.-A. Bulzu, H. L. Banciu, A. Ştefan Andrei, T. Uchihashi, R. Ghai, O. Béjà and H. Kandori, *Sci. Adv.*, 2020, **6**, eaaz2441.

[6] L. Pedraza-González, L. Barneschi, M. Marszałek, D. Padula, L. De Vico and M. Olivucci, *J. Chem. Theory Comput.*, 2022, **19**, 293–310.

[7] L. Barneschi, E. Marsili, L. Pedraza-González, D. Padula, L. De Vico, D. Kaliakin, A. Blanco-González, N. Ferré, M. Huix-Rotllant, M. Filatov and M. Olivucci, *Nat. Commun.*, 2022, **13**, 6432.

[8] L. Pedraza-González, L. De Vico, M. d. C. Marın, F. Fanelli and M. Olivucci, *J. Chem. Theory Comput.*, 2019, **15**, 3134–3152.

[9] J. F. Bada Juarez, P. J. Judge, S. Adam, D. Axford, J. Vinals, J. Birch, T. O. Kwan, K. K. Hoi, H.-Y. Yen, A. Vial *et al.*, *Nat. Commun.*, 2021, **12**, 629.

[10] R. Palombo, L. Barneschi, L. Pedraza-González, X. Yang and M. Olivucci, *Phys. Chem. Chem. Phys.*, 2024, **26**, 10343–10356.

[11] S. Battaglia and R. Lindh, *J. Chem. Theory Comput.*, 2020, **16**, 1555–1567.

[12] Y. Nishimoto, S. Battaglia and R. Lindh, *J. Chem. Theory Comput.*, 2022, **18**, 4269–4281.

[13] A. Penzkofer, A. Silapetere and P. Hegemann, *International Journal of Molecular Sciences*, 2019, **20**, 4086.

[14] W. D. Cornell, P. Cieplak, C. I. Bayly, I. R. Gould, K. M. Merz, D. M. Ferguson, D. C. Spellmeyer, T. Fox, J. W. Caldwell and P. A. Kollman, *J. Am. Chem. Soc.*, 1996, **118**, 2309–2309.

[15] N. Ferré, A. Cembran, M. Garavelli and M. Olivucci, *Theoretical Chemistry Accounts*, 2004, **112**, 335–341.

[16] E. Marsili, M. Olivucci, D. Lauvergnat and F. Agostini, *J. Chem. Theory Comput.*, 2020, **16**, 6032–6048.

[17] F. Aquilante, J. Autschbach, R. K. Carlson, L. F. Chibotaru, M. G. Delcey, L. De Vico, I. Fdez. Galván, N. Ferré, L. M. Frutos, L. Gagliardi *et al.*, *Molcas 8: New capabilities for multiconfigurational quantum chemical calculations across the periodic table*, 2016.

[18] J. A. Rackers, Z. Wang, C. Lu, M. L. Laury, L. Lagardère, M. J. Schnieders, J.-P. Piquemal, P. Ren and J. W. Ponder, *J. Chem. Theory Comput.*, 2018, **14**, 5273–5289.

[19] J. C. Tully, *J. Chem. Phys.*, 1990, **93**, 1061–1071.

[20] G. Granucci and M. Persico, *J. Chem. Phys.*, 2007, **126**, year.

[21] M. Manathunga, X. Yang, H. L. Luk, S. Gozem, L. M. Frutos, A. Valentini, N. Ferrè and M. Olivucci, *J. Chem. Theory Comput.*, 2016, **12**, 839–850.

[22] L. Barneschi, D. Kaliakin, M. Huix-Rotllant, N. Ferré, M. Filatov and M. Olivucci, *J. Chem. Theory Comput.*, 2023, **19**, 8189–8200.

[23] S. Gozem, H. L. Luk, I. Schapiro and M. Olivucci, *Chem. Rev.*, 2017, **117**, 13502–13565.

[24] L. M. Frutos, T. Andruniów, F. Santoro, N. Ferré and M. Olivucci, *Proc. Natl. Acad. Sci. U.S.A.*, 2007, **104**, 7764–7769.
